# Carborane-based BODIPY dyes: synthesis, structural analysis, photophysics and applications

**DOI:** 10.3389/fchem.2024.1485301

**Published:** 2024-11-05

**Authors:** Javier Ordóñez-Hernández, José Giner Planas, Rosario Núñez

**Affiliations:** Institut de Ciència de Materials de Barcelona (ICMAB-CSIC), Bellaterra, Spain

**Keywords:** BODIPY, carboranes, boron clusters, luminescence, bioimaging, fluorescence, FRET, BNCT

## Abstract

Icosahedral boron clusters-based BODIPY dyes represent a cutting-edge class of compounds that merge the unique properties of boron clusters with the exceptional fluorescence characteristics of BODIPY dyes. These kinds of molecules have garnered substantial interest due to their potential applications across various fields, mainly including optoelectronics, bioimaging, and potential use as boron carriers for Boron Neutron Capture Therapy (BNCT). Carborane clusters are known for their exceptional stability, rigid geometry, and 3D-aromaticity, while BODIPY dyes are renowned for their strong absorption, high fluorescence quantum yields, and photostability. The integration of carborane into BODIPY structures leverages the stability and versatility of carboranes while enhancing the photophysical properties of BODIPY-based fluorophores. This review explores the synthesis and structural diversity of boron clusters-based BODIPY dyes, highlighting how carborane incorporation can lead to significant changes in the electronic and optical properties of the dyes. We discuss the enhanced photophysical characteristics, such as red-shifted absorption and emission poperties, charge and electronic transfer effects, and improved cellular uptake, resulting from carborane substitution. The review also delves into the diverse applications of these compounds. In bioimaging, carborane-BODIPY dyes offer superior fluorescence properties and cellular internalization, making them ideal for cell tracking. In photodynamic therapy, (PDT) these dyes can act as potent photosensitizers capable of generating reactive oxygen species (ROS) for targeted cancer treatment making them excellent candidates for PDT. Additionally, their unique electronic properties make them suitable candidates for optoelectronic applications, including organic light-emitting diodes (OLEDs) and sensors. Overall, carborane-BODIPY dyes represent a versatile and promising class of materials with significant potential for innovation in scientific and technological applications. This review aims to provide a comprehensive overview of the current state of research on carborane-BODIPY dyes, highlighting their synthesis, properties, and broad application spectrum.

## Graphical Abstract

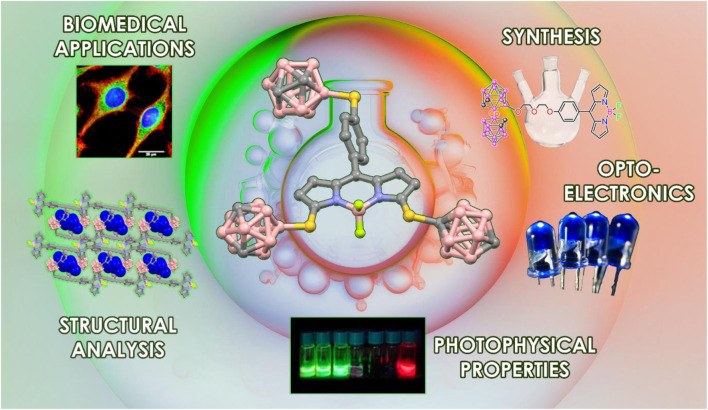

## 1 Introduction

**CHART 1:**
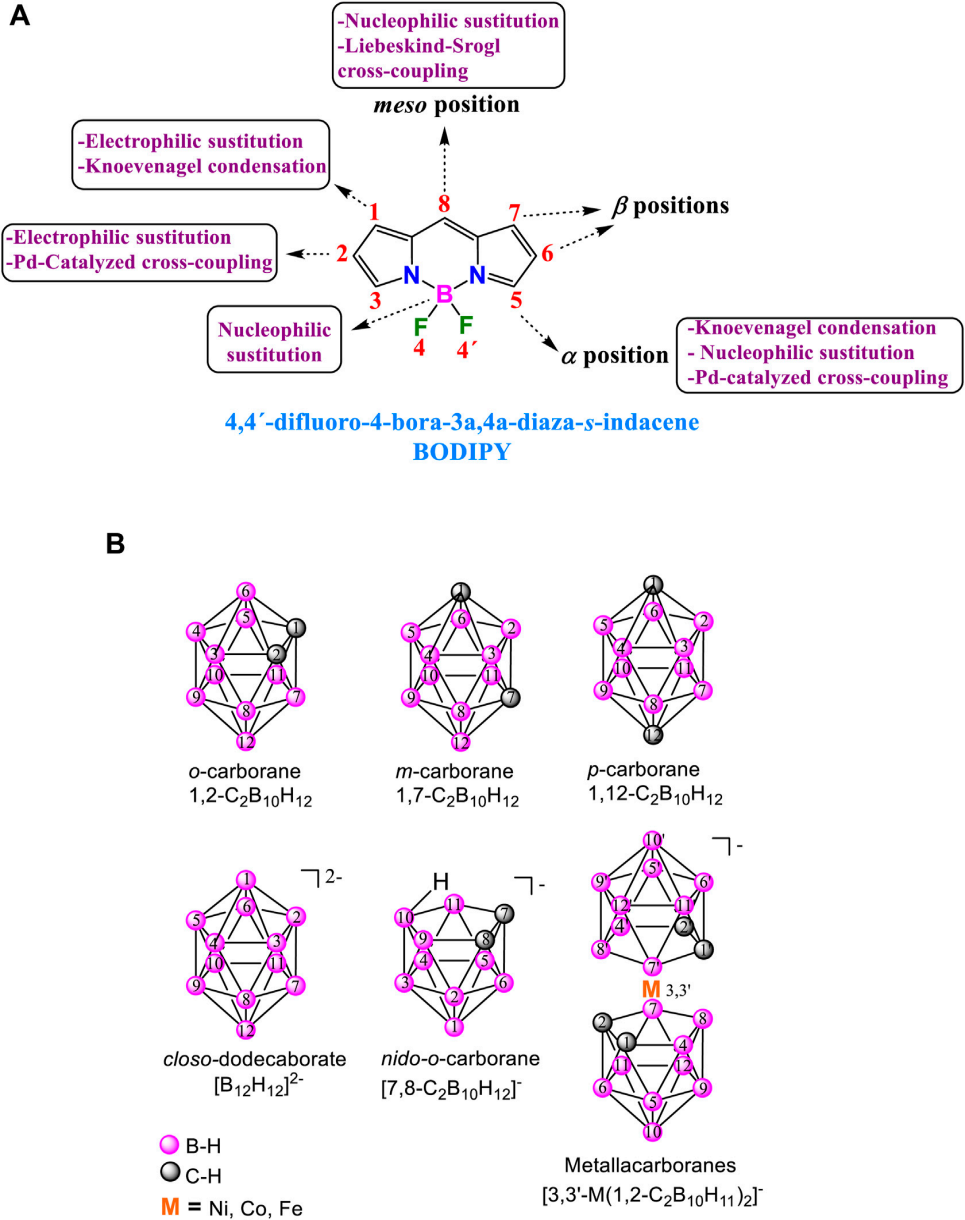
**(A)** Structure of BODIPY, IUPAC and common nomenclature and main accessible routes for the functionalization. **(B)** (Top) Isomeric neutral icosahedral boron clusters: *o*-carborane, *m*-carborane and *p*-carborane. (Bottom) Anionic boron clusters with their vertexes numbering.

Among the vast array of known fluorescent dyes, the 4,4-difluoro-4-bora-3a,4a-diaza-s-indacene more known as boro-dipyrromethene (abbreviated as BODIPY; Chart 1) has emerged as one of the most versatile reagents and has garnered popularity among the scientific community. BODIPYs have intrinsic unique properties, including strong electron donating properties, excellent chemical, thermal and optical stability, high molar absorptivity and quantum yield (QY or φ_F_) and versatility for their functionalization at different positions. BODIPY dyes show a strong and broad absorption band at around 500–550 nm, corresponding to the S_0_-S_1_ transition (π-π*) with high molar extinction coefficients (4000–110000 M^−1^cm^−1^), emit a narrow spectrum between 530–560 nm and present high fluorescence QY. Additionally, they exhibit thermal and photochemical stability and good solubility in various organic solvents; some derivatives are also soluble in water.

These dyes are relatively easy to synthesize, and various methods can be employed to modify the BODIPY core at the α, β, *meso* position, and at the boron atom employing adequate conditions (Chart 1A). (Loudet and Burgess, 2007) These methods encompass Knoevenagel-type condensation, nucleophilic and electrophilic substitutions, Pd-catalyzed C-C cross-coupling reactions, Liebeskind-Srogl cross-coupling, Grignard reactions, direct styrylation, replacement of fluorine atoms at the boron centre, etc. All these synthetic techniques can be utilized to synthesize BODIPY derivatives, allowing for precise adjustment of their photonic and electronic properties. Due to all the characteristics described above, in the last 3 decades, a significant number of BODIPY derivatives have been designed and synthesized for their use in material science and biomedical applications (Li et al., 2024; Das et al., 2023), such as fluorescent switches, electroluminescent films, laser dyes (Avellanal-Zaballa et al., 2022), energy transfer cassettes, light harvesting, triplet photosensitizers (for solar cells, organo photocatalysis and of singlet oxygen production for photodynamic therapy) (Yadav and Misra, 2023), nonlinear optics, bioimaging, and chemosensing (Bumagina and Antina, 2024).

Carboranes are a unique class of inorganic polyhedral clusters composed primarily of boron, carbon, and hydrogen atoms. Their distinct structural characteristics and remarkable chemical stability have garnered significant interest across various fields of chemistry and materials science. Among the carborane cluster families, the twelve-vertex icosahedral dicarba-*closo*-dodecaborane, also known as *closo*-carborane (C_2_B_10_H_12_), and its derivatives are the most extensively studied (Chart 1B). (Grimes, 2016; Scholz and Hey-Hawkins, 2011), These clusters, considered analogues to arenes, show three dimensional (3D) geometry and σ-aromaticity (Poater et al., 2014; Poater et al., 2020). The *ortho*-carborane (*o*-carborane, 1,2-C_2_B_10_H_12_) can be thermally isomerized to produce *meta*-carborane (*m*-carborane, 1,7-C_2_B_10_H_12_) (Chart 1B) and finally, *para*-carborane (*p*-carborane, 1,12-C_2_B_10_H_12_) (Grimes, 2016). These neutral carboranes are remarkably robust, with unique properties such as hydrophobicity, electronic and thermal stability, chemical versatility to be functinalized both at C and B atoms of the cluster) and low toxicity in biological systems. Moreover, the *closo-o*-carborane can be partially deboronated in basic media, by removing formally a BH^+^ vertex to give the corresponding anionic species *nido-o*-carborane (Chart 1B). Deprotonation of *nido*-*o*-carborane and further reaction with transition metal salts (mainly NiCl_2_, CoCl_2_ and FeCl_2_) led to the formation of anionic θ-shape metallacarboranes (Chart 1B). These metallacarboranes have high molecular volume, low nucleophilic character, low charge densities, as well as extraordinary high chemical, thermal and photochemical stability, and excellent biocompatibility (Housecroft, 2011). All these properties make these boron clusters promising building blocks for a wide variety of applications (Fisher et al., 2019; Sivaev, I. B. and Bregadze, 2019) such as optoelectronic materials (Mukherjee and Thilagar, 2016; Núñez et al., 2016b; Ochi et al., 2020; Parejo et al., 2021), polymers (Núñez et al., 2016a), and biomedical applications (Valliant et al., 2002; Sivaev and Bregadze, 2009; Issa et al., 2011; Vinas, 2013; Leśnikowski, 2016; Stockmann et al., 2019), among others.

In recent years, the luminescent properties of carborane-containing compounds have become a focal point of research, particularly due to their potential applications in optoelectronics, sensing, and bioimaging. The incorporation of carborane moieties into organic and inorganic frameworks can profoundly influence the photophysical properties of the resulting materials. This influence is attributed to the electron-deficient nature of boron atoms within the carborane cluster, which can modulate electronic transitions and enhance luminescent efficiency. Regarding the electronic effect, *o*-carborane behaves as a strong electron-withdrawing group (similar to fluorinated aryl) on a substituent at one of the cluster carbons (C_cluster_, C_c_). When *o*-carborane is linked to an aromatic donor group, the intramolecular charge transfer (ICT) process occurs from the donor moiety to the acceptor boron cluster, which is influenced by the C_c_-C_c_ bond vibration (Oliva et al., 2005), usually producing the quenching of the fluorescence in solution. Moreover, carboranes are known to impart unique photophysical behaviors such as aggregation-induced emission (AIE) and thermally activated delayed fluorescence (TADF) (Ochi et al., 2020). These phenomena are of considerable interest for developing advanced luminescent materials. The rigidity and electron-withdrawing nature of carboranes stabilize excited states, thereby extending emission lifetimes and improving QYs in aggregate and solid state (Naito et al., 2017; Tu et al., 2019; Wei et al., 2019; Ochi et al., 2020; Cabrera-Gonzalez et al., 2016; 2017; Chaari et al., 2018b; 2019; 2020; Cabrera-González et al., 2020).

**CHART 2:**
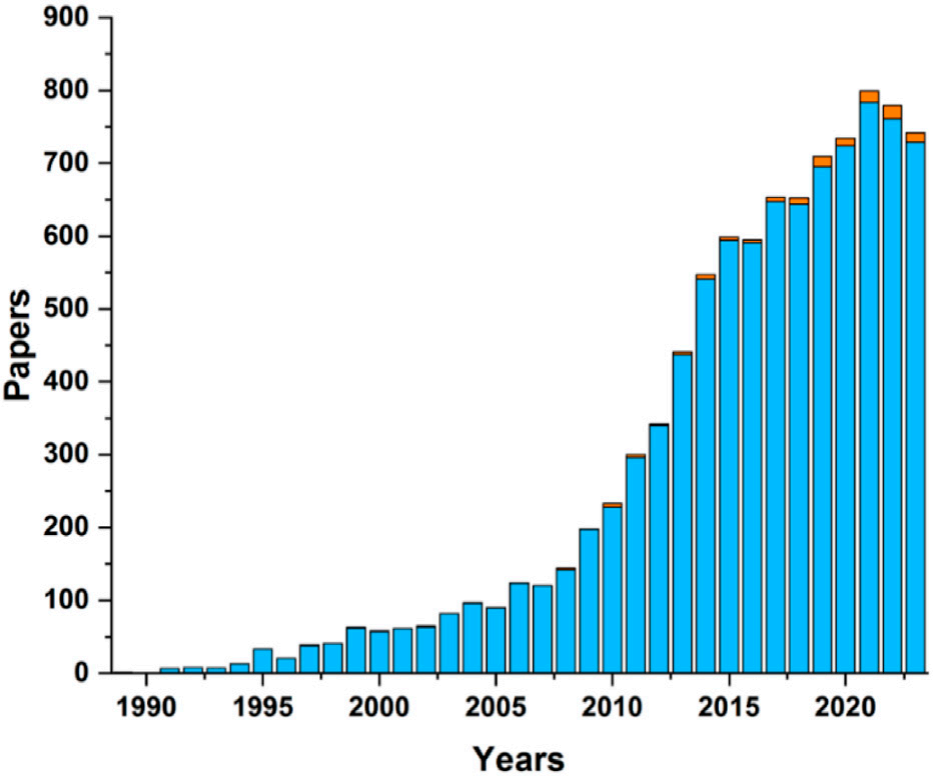
Number of publications of BODIPYs (blue bars) and BODIPYs containing boron clusters (orange bars) since 1990. The data was taken from the Web of Science.

Carborane-BODIPY dyes represent an innovative class of luminescent compounds that uniquely combine the exceptional properties of boron-rich carborane clusters with the renowned fluorescence characteristics of BODIPY fluorophores. Carborane clusters, known for their chemical stability and electron-withdrawing capabilities, provide a robust and versatile framework that significantly enhances the optical and electronic properties of BODIPY dyes. The photophysical behavior of these carborane-based BODIPY dyes is primarily shaped by the strong electronic interactions between the carborane cluster and the BODIPY core. These interactions often result in notable modifications to the absorption and emission spectra, most prominently inducing a red-shift (bathochromic shift) in the fluorescence emission. This shift is attributed to the stabilization of the lowest unoccupied molecular orbital (LUMO) by the electron-withdrawing effect of the carborane unit, thereby narrowing the HOMO-LUMO energy gap. The integration of carborane clusters not only enhances fluorescence but also improves the overall chemical stability and bio-compatibility of the molecules, making them highly promising for a range of cutting-edge applications, particularly in biomedicine, where targeted imaging and therapy are crucial, as well as in advanced optoelectronic devices. Despite the interesting synergy in the properties of both fragments, BODIPY and boron clusters, the small number of examples of these systems in the literature is surprising as shown in graph depicted in Chart 2.

This review aims to describe the various synthetic routes for incorporating different boron clusters (carboranes and metallacarboranes) into BODIPY cores, delve into the mechanisms by which carborane clusters affect the photoluminescence properties, explore recent advancements in luminescent carborane-based BODIPY dyes, and highlight their potential applications. By understanding the interplay between carborane structure and luminescent properties, we can pave the way for materials with tailored photophysical characteristics for cutting-edge technological applications. The review is organized into several sections, classifying carborane-BODIPY dyes based on their properties and potential applications. Most of the BODIPYs featuring boron clusters identified in the literature can be gathered into two main categories. The first category includes BODIPY systems with notable photophysical properties, in which the presence of the boron cluster can influence the fluorescence resonance energy transfer or Förster resonance energy transfer (FRET), photoinduced electron transfer (PET), or AIE processes. In this category we have also included nanocar-type systems suitable for single-molecule fluorescence microscopy (SMFM) experiments. The second large section includes those designed boron cluster containing BODIPY for biological and biomedical applications, mainly *in vitro* bioimaging and Boron Neutron Capture Therapy (BNCT).

## 2 Boron cluster-based BODIPY dyes for promising optoelectronic applications

### 2.1 Spatial energy transfer in BODIPY-Based donor-acceptor dyads via carborane linkers

**SCHEME 1:**
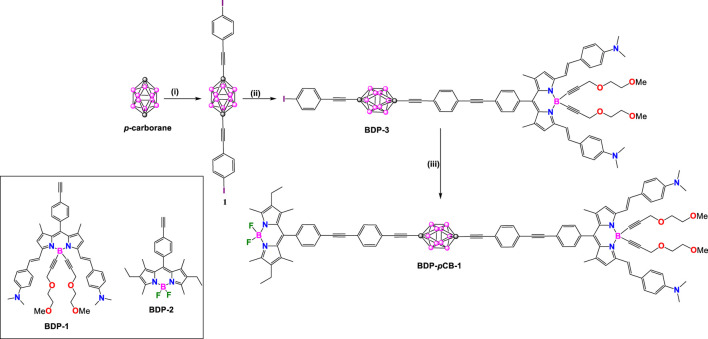
Synthesis of **BDP-*p*CB-1.** Steps: (i) *n*BuLi, THF, −78°C, CuBr, 4-(bromoethynyl)iodobenzene, 35%; (ii) **BDP-1**, benzene, triethylamine, [Pd(PPh_3_)_4_], 60°C, overnight, 33%; (iii) **BDP-2**, benzene, Et_3_N, [Pd(PPh_3_)_4_], 60°C, 36 h, 70%.

Ziessel and coworkers synthesized a series of fluorescent dyads using a *p*-carborane as a linker between different fluorophores, with one acting as the electron-donor group and the other as the electron-acceptor group (Scheme 1). These systems demonstrated spatial energy transfer through the carborane linker making them promising candidates for optoelectronic devices, sensors, data storage or data computing.

In 2010, They synthesized a rigid molecular donor-acceptor dyad, **BDP-pCB-1**, arranged in a rod-like structure using a 1,12-dicarba-*closo*-dodecaborane (*p*-carborane) core to link two distinct BODIPY modules. This design was intended to facilitate through-space energy transfer (Ziessel et al., 2010). The target molecule **BDP-*p*CB-1** was synthesized by metalation of *p*-carborane employing *n*-BuLi and CuBr under kinetical control followed by adding 4-(bromoethynyl)iodobenzene to provide the disubstituted compound **1**. Taking advantage of the presence of two iodo groups at the vertices of compound **1**, it is possible to link ethynyl-substituted BODIPY dyes by a sequence of two Sonogashira cross-coupling reactions to yield rigid dyad **BDP-*p*CB-1**.

**SCHEME 2:**
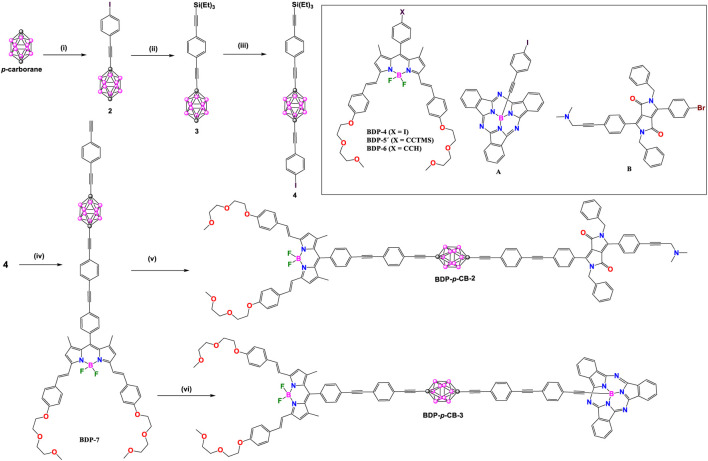
Steps: (i) *n*BuLi, CuBr, 4-(bromoethynyl)iodobenzene; (ii) thiethylsilylacetylene, [Pd(PPh_3_)_2_Cl_2_]/CuI, 92%; (iii) *n*BuLi, CuBr, 4-(bromoethynyl)iodobenzene; (iv) 1) [Pd(PPh_3_)_4_]/CuI, compound **BDP-6**, 93%, 2) KF, 92%; (v) [Pd(PPh_3_)_4_]/CuI, compound **B**, 85%, (vi) [Pd(PPh_3_)_4_]/CuI, compound **A**, 86%.

**SCHEME 3:**
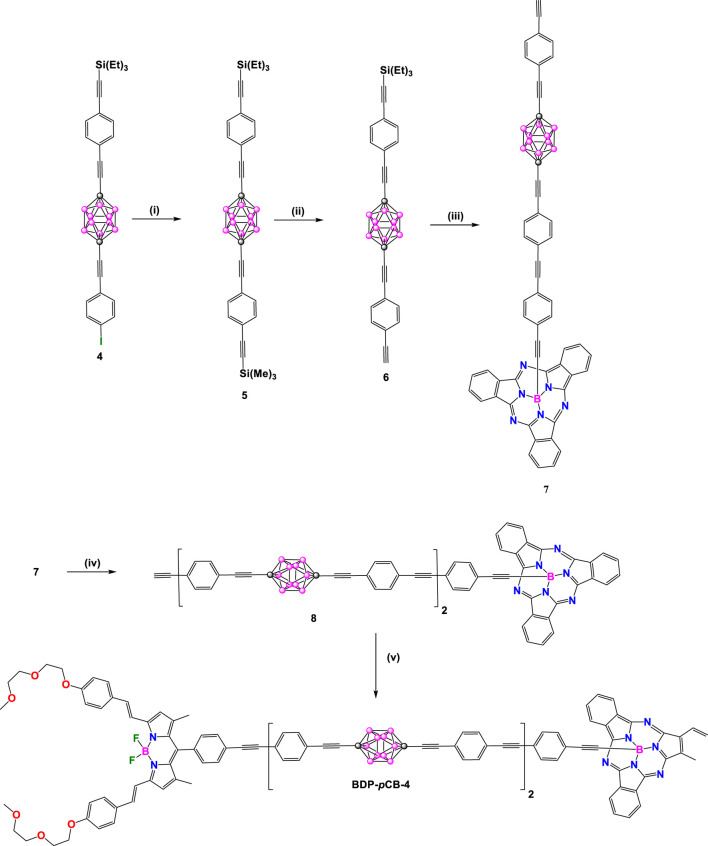
Steps: (i) trimethylsilylacetylene, [Pd(PPh_3_)_2_Cl_2_]/CuI, 91%; (ii) K_2_CO_3_ (1 equiv.), rt, 86%; (iii) [Pd(PPh_3_)_4_], triethylamine, compound **A**, 86%; (iv) 1) KF, 50%, 2) [Pd(PPh_3_)_4_], Et_3_N, compound **7**, 78% 3) KF, 52% (v) [Pd(PPh_3_)_4_], compound **BDP-4**, 78%.

Two years later, unsymmetrical **BDP-*p*CB-2**-**4** derivatives were synthesized by the same authors (Schemes 2, 3). In these donor-acceptor dyads, the BODIPY (λ_abs_/λ_em_ at 645/659 nm) was chosen as acceptor unit and subphthalocyanine (SubPc: λ_abs_/λ_em_ at 565/574 nm) in **BDP-*p*CB-2** and diketopyrrolopyrrole (DPP: λ_abs_/λ_em_ at 645/659 nm) in **BDP-*p*CB**-**3** and **BDP-*p*CB-4** as donor cores (Hablot et al., 2012). Dyads **BDP-*p*CB-2** and **BDP-*p*CB-3** were obtained from common intermediate **4** (Scheme 2); firstly, bonding the **BDP-6** with **4** by Sonogashira cross-coupling reaction followed by deprotection of triethylsilyl (TES) group with KF to yield **BDP-7**. Finally, compound **BDP-7** was linked to a second fluorophore, such as **A** or **B**, by other Sonoghashira reactions to produce the dyads **BDP-*p*CB-2** and **BDP-*p*CB-3**. Additionally, dyad **BDP-*p*CB**-**4** with two *p*-carborane linkers between BODIPY and SubPc cores was prepared through a sequence of Pd-catalyzed couplings (Scheme 3), to compare the effect of increasing the length of the spacing unit on the efficiency of electronic energy transfer between the donor to the acceptor systems.

**SCHEME 4:**
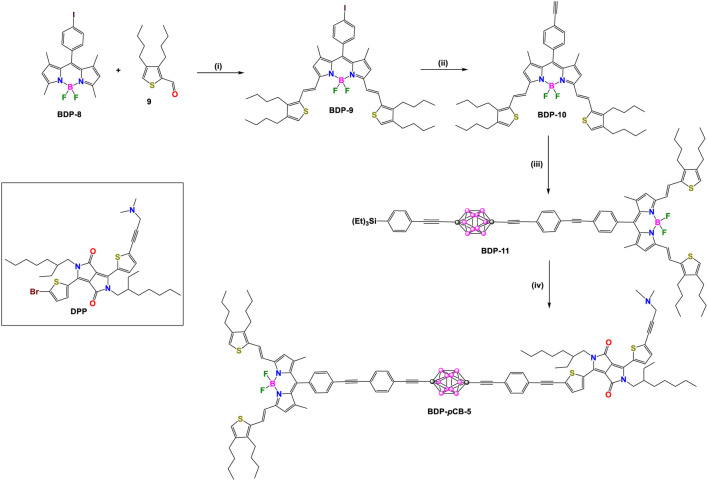
Steps: (i) piperidine, *p*TSOH cat., toluene, reflux, 52%; (ii) 1) [Pd(PPh_3_)_2_Cl_2_], CuI, trimethylsilylacetylene, benzene/Et3N, 60°C, 97%, 2) K_2_CO_3_, THF/MeOH/H_2_O, room temperature (r.t.), 94%. (iii) **4**, [Pd(PPh_3_)_4_], benzene/Et_3_N, 60°C, 97%; (iv) 1) NaOH/H_2_O, 60°C, 79%; 2) **DPP**, [Pd(PPh_3_)_4_], benzene/Et_3_N, 60°C, 78%.

**SCHEME 5:**
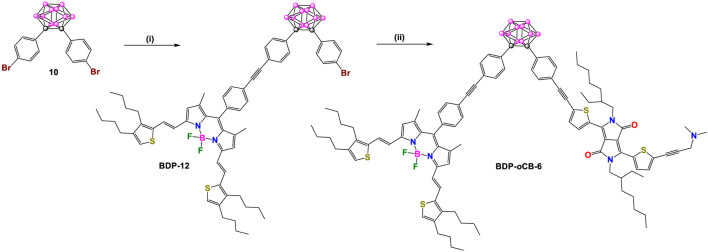
Steps: (i) **BDP-10**, [Pd(PPh_3_)_4_], benzene/Et_3_N, 60°C, 72%; (ii) 1) [Pd(PPh_3_)_2_Cl_2_], CuI, trimethylsilylacetylene, benzene/Et_3_N, 50°C, 84%, 2) K_2_CO_3_, THF/MeOH/H_2_O, r.t, 92%; 3) **DPP**, benzene/Et_3_N, 60°C, 84%.

In 2013, Ziessel, Harriman, and coworkers reported two other donor-acceptor dyads, **BDP-*p*CB-5** and **BDP-*o*CB-6** (Schemes 4, 5), employing *para-* and *ortho-*carborane as linkers, respectively (Harriman et al., 2013). In each compound, the acceptor unit is a BODIPY (BDP: λ_abs_/λ_em_ at 672/687 nm) conjugated to two ethenylthiophene groups, and the donor unit is a diketopyrrolopyrrole (DPP: λ_abs_/λ_em_ at 605/627 nm). Detailed synthetic steps for synthesizing these dyads are shown in Schemes 4, 5, respectively. Intermediate **BDP-10** was synthesized in three steps. Firstly, a Knoevenagel-type condensation reaction between **BDP-8** and thiophene derivative **9** gives **BDP-9**. Reaction of **BDP-9** with trimethylsilylacetylene under Pd-catalyzed cross-coupling reaction conditions followed by subsequent removal of the trimethylsilane (TMS) group yielded **BDP-10.** The rest of the synthesis routes to obtain dyads **BDP-*p*CB-5** and **BDP-*o*CB-6** followed the same strategy based on Pd-catalyzed cross-coupling reactions between pre-functionalized carborane clusters and fluorophores.

**SCHEME 6:**
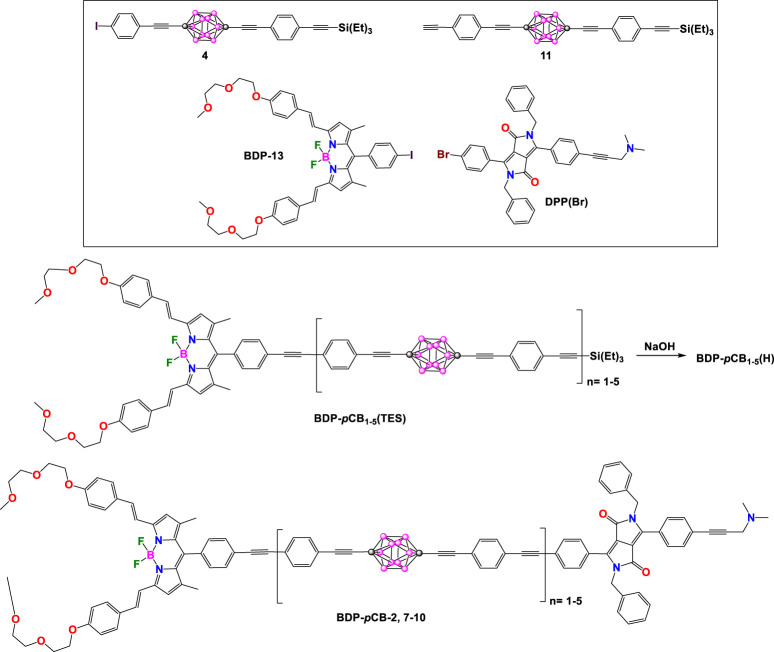
Chemical structures of the building blocks required to prepare **BDP-*p*CB-2**, **BDP-*p*CB-7-10**.

The same year, Ziessel and Harriman reported a series of linear donor-spacer acceptor compounds, including the previously reported **BDP-*p*CB**-2 and new dyads **BDP-*p*CB-**7, BDP-pCB-8, BDP-pCB-9 and BDP-pCB-10 (Scheme 6) (Hablot et al., 2013). The spacer was built by increasing of *p*-ethynylene-carborane units, which provided acceptor-donor separation distances of 38, 57, 76, 96, and 115 Å along the series. All dyads were synthesized using the building blocks shown in Scheme 6, following an iterative synthetic protocol. The first step involves a Pd-catalyzed cross-coupling reaction between **BDP-13** and compound **11**. Deprotection of the triethysilyl group using NaOH as base afforded the **BDP-*p*CB(H)** cross-linking to **DPP(Br)** to obtain **BDP-*p*CB-8** with n = 1. **BDP-*p*CB(H)** was coupled to a second compound **4** and then deprotected and involved in further cross-linking with **DPP(Br)** yielding dyad **BDP-*p*CB-7** with n = 2. The rest of the dyads **BDP-*p*CB9** and **BDP-*p*CB-10** were prepared following the same strategy using **BDP-*p*CB(H)** as a critical component to extend the bridge.

In **BDP-*p*CB-1**, the **BDP-1** core acts as an electron donor unit; it contains two dimethylamino groups sensitive to pH, enabling variation of the spectral overlap to the donor module, **BDP-2** core acts as an electron acceptor unit, and *p*-carborane has no significant electronic influence. Optical data for all compounds in this section are summarized in Supplementary Table S1. Protonation of **BDP-*p*CB-1** (in dioxane) with HCl (g) induces a strong hypsochromic shift, shifting the absorption band from 694 nm to 628 nm and the emission band from 728 to 638 nm, while increasing the QY from 30% to 42%. Remarkably, the absorption and emission spectra of **BDP-*p*CB-1** in its deprotonated and protonated forms are linear combinations of the absorption and emission of **BDP-1** (deprotonated and protonated) and **BDP-2** (λ_abs_ = 525 nm and λ_em_ = 538 nm). Förster Resonance Energy Transfer (FRET) efficiency between two BODIPY modules in dyad **BDP-*p*CB-1** in its deprotonated and protonated forms was calculated from measured fluorescence QYs to be 82% and 89%, with constant for FRET of 6.2 × 10^8^ and 1.7 × 10^9^ S^−1^ respectively, and a center D-A distance of 36.8 Å.

Compounds **BDP-*p*CB 2**, **BDP-*p*CB 3** and **BDP-*p*CB-4** exhibited absorption and emission spectra show the bands typical of each photoactive module with no significant influence of the *p*-carborane linker (Supplementary Table S1). Dyads **BDP-*p*CB-2** and **BDP-*p*CB-3** present a very efficient electronic energy transfer (EET>99%) with rate constants calculated for EET of 1.2 × 10^10^ s^−1^ and 1.7 × 10^9^ s^−1^, respectively. Compound **BDP-*p*CB-4** reaches 76% of EET with a rate constant of 3.2 × 108 s^−1^, indicating that increasing the distance between donor-acceptor groups decreases EET efficiency.

The excitation spectra confirmed that intramolecular EET occurs from DPP to BODIPY on **BDP-*p*CB5** and **BDP-*o*CB6** dyads. The donor’s emission spectrum and the acceptor’s absorption spectrum showed excellent spectral overlap, resulting in high efficiency for EET. BDP-pCB-5 has a KEET of 3.1 ± 0.2 × 109 s^−1^ and an efficiency EET process >90% with a donor-acceptor distance of 36 Å. The dyad BDP-oCB-6 has a KEET of 1.9 ± 0.2 × 1010 s^−1^ and an efficiency EET process >95% with a donor-acceptor distance of 19 Å. In line with the previous report, it is worth noting that the EET efficiency and the KET increased when the acceptor and the donor units were closer to each other.

In compounds **BDP-*p*CB-2** and **BDP-*p*CB-7-10**, the intensity of both absorption and emission as well as fluorescent QY increase with the length of dyads (Supplementary Table S1). It is noteworthy that **BDP-*p*CB-2** produces poor fluorescent QY (0.09), which is much lower than the larger dyads (QY = 0.44, 0.73, 0.81, and 0.84). Computational calculations for all dyads established that the Förster critical distance of the two isolated chromophores is 54.4 Å at room temperature and 52.8 Å at 77 K, which supports the experimental data. The efficiency parameters of EET between acceptor-donor units depend on the length of the spacer and external factors such as temperature or pressure. As the temperature decreases (spectra recorded in MTHF), the emission maxima band of the BODIPY acceptor is red-shifted while the emission maxima band of the acceptor is blue-shifted; consequently, there is a progressive decrease in the integral of the spectral overlap, and therefore it decreases EET efficiency from DPP to BODIPY. Additionally, when pressure increases, there is a decrease in the fluorescence of BODIPY and an increase in the fluorescence of the DPP, which reduces the probabilities of EET.

### 2.2 Fluorescence resonance energy transfer in donor-acceptor dyads via metallacarborane linkers

**SCHEME 7:**
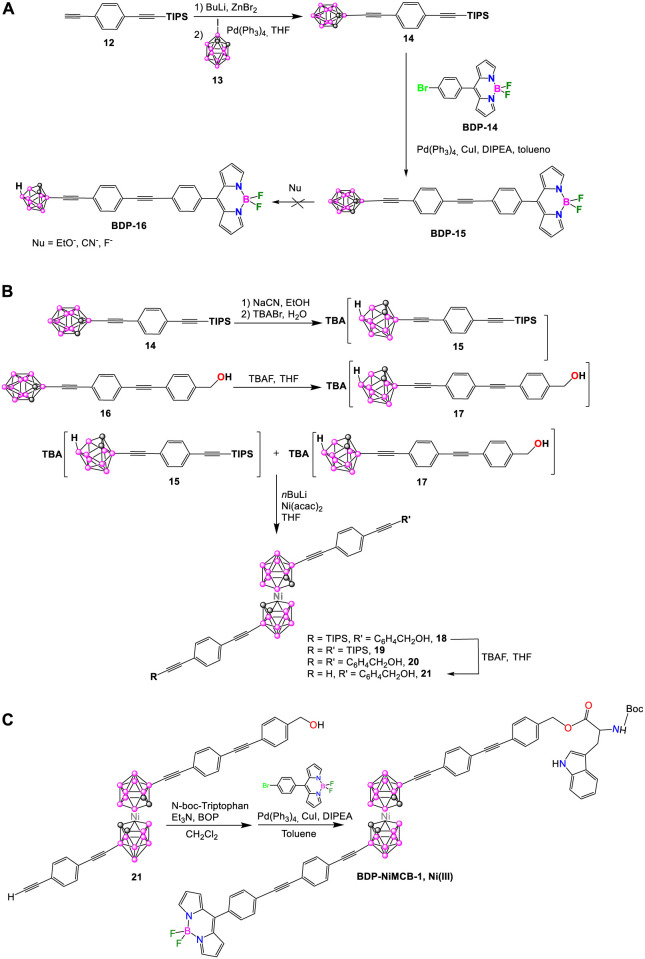
**(A)** Reaction to attempt the synthesis of **BDP-16** containing *nido-o*-carborane. **(B, C)** Synthetic procedure for the metallacarborane-based BODIPY **BDP-NiMCB-1**.

In 2015, Hawthorne et al. reported the synthesis of a nickelacarborane complex (**BDP-NiMCB-1**, Scheme 7) bearing two fluorophore molecules capable of Förster resonance energy transfer (FRET). In this dye, the *N*-boc-tryptophan and 8-(40-bromophenyl)-4,4-difluoro-4-bora-3a, 4a-diaza-*s*-indacene were chosen as the FRET donor and acceptor groups, respectively (Shlyakhtina et al., 2015). The first attempt to prepare **BDP-NiMCB-1** was through the synthesis of **BDP-16** upon deboronation reaction from **BDP-15** using different nucleophiles (Scheme 7A), however only traces of the target **BDP-16** were obtained. The second synthetic strategy is presented in Scheme 7B, C. Successful deboronation of compounds **14** and **16** led to the formation of the corresponding *nido*-carborane derivatives **15** and **17** (Scheme 7B). The synthesis of the mixed-ligand Ni(III) nickelacarborane **18** was carried out by the reaction of stoichiometric amounts of tetrabutylammonium salts of *nido*-carboranes **15** and **17** with excess butyllithium and then with Ni(acac)_2_ in THF (Scheme 7B). After a complicated separation of complexes **18**, **19,** and **20** by chromatography, paramagnetic compound **21** was formed in excellent yield (92%) by deprotonation reaction of **18** with TBAF in THF (Scheme 7B). The reaction of **21** with previously esterified *N*-boc-triptophan in the presence of Et_3_N in dichloromethane (DCM) gave the corresponding intermediate in 76% yield (Scheme 7C). The final step was the Sonogashira cross-coupling reaction of the intermediate with **BDP-14** in the presence of Pd(PPh_3_)_4_ catalyst to give **BDP-NiMCB-1** in 5.5% yield (Scheme 7C).

The absorption spectrum of **BDP-NiMCB-1** showed characteristic bands for all parts of the molecules. The absorption band centered at around 300 nm corresponds to tryptophan and the linker, whereas the maximum of absorption at 500 nm and the broad band in the 350–400 nm region corresponds to the BODIPY. The emission spectrum showed a maximum centered at 532 nm, a low-intensity band at 345 nm, attributed to the residual fluorescence of tryptophan, and a broad emission in the range 450–500 nm, with difficult assignation. The presence of the BODIPY emission band at 532 nm can only be explained by FRET in solution.

### 2.3 Photoinduced electron transfer in carborane-BODIPY dye systems

The development of electron donor-acceptor systems that exhibit photoinduced electron transfer (PET) have attracted significant attention in many areas, including charge separation processes in photovoltaics, molecular electronics, light-emitting diodes, photoluminescence sensors and switches. In solar energy conversion and storage, and photovoltaics. Low-energy photosensitization using electron donor-acceptor dyads is crucial for efficient solar light harvesting in artificial photosynthesis systems and solar cells (Kulinich and Ishchenko, 2024; Tan et al., 2023; Imahori and Akiyama, 2024; Feng et al., 2023). In the following section, we will describe a series of BODIPY functionalized with carborane derivatives that exhibited photoinduced electron transfer (PET) processes.

**SCHEME 8:**
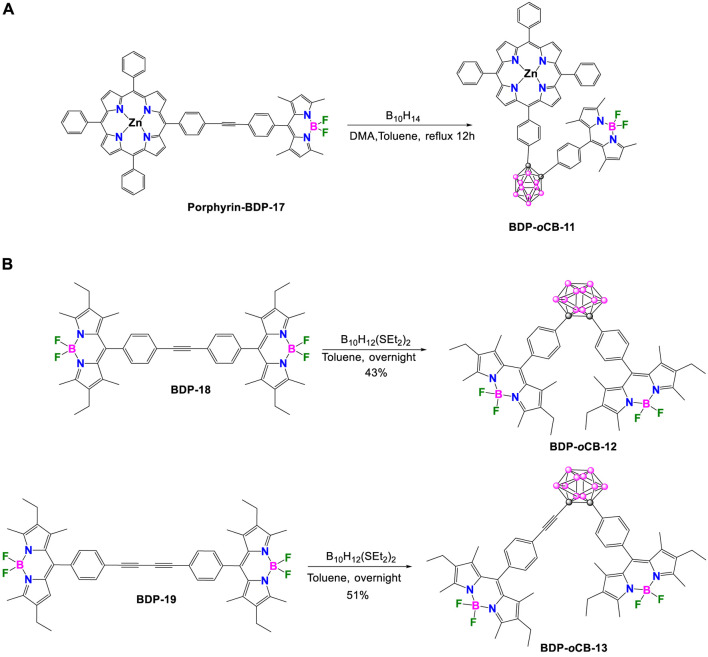
**(A)** Synthesis of the triad **BDP-*o*CB-11. (B)** Synthesis of **BDP-*o*CB-12** and **BDP-*o*CB-13**.

In 2018, E. Hamuryudan et al. (Berksun et al., 2018) synthesized the porphyrin–*o*-carborane–BODIPY triad **BDP-*o*CB-11** by the reaction of a previously prepared **porphyrin-BDP-17** dyad with decaborane (B_10_H_14_), using *N*,*N*-dimethylaniline in toluene at reflux for 12 h. After extraction and purification by column chromatography on silica gel, hexane/DCM (2:1, v/v) was used as the eluent, and compound **BDP-*o*CB-11** was obtained in 46% yield (Scheme 8A).

The UV-Vis spectrum of the compound **BDP-*o*CB-11** in toluene showed two separated bands at 424 and 506 nm, which correspond to the absorption bands of the porphyrin and BODIPY fragments, respectively (Berksun et al., 2018). The spectrum of the compound was very similar to their independent precursors, the BODIPY and the porphyrin, which suggested that the presence of the carborane as a linker between both fluorophores does not affect the electronic properties of the triad, and there is no significant ground state interaction between the fluorophores. **BDP-*o*CB-11** exhibited weak fluorescence in THF with low quantum yield of 0.028 that was attributed to the quenching caused by the *o*-carborane. Due to the electron-withdrawing character of the *o*-carborane through the C_c_ (Núñez et al., 2006), when the porphyrin or the BODIPY in the **BDP-*o*CB-11** were selectively excited, an efficient PET occurs through the antibonding orbitals of the C–C bonds of the *o*-carborane, producing the quenching of the **BDP-*o*CB-11**. When the BODIPY unit in triad **BDP-*o*CB-11** was selectively excited, was observed a resonance energy transfer (RET) to the corresponding porphyrin unit. However, it was not possible to calculate the energy transfer efficiency for the triad, as the fluorescence of the donor unit was almost completely quenched, due to the PET process in the presence of *o*-carborane.

In 2015, Kang and coworkers reported on two V-shaped triads, **BDP-*o*CB-12** and **BDP-*o*CB-13**, designed for low-energy photosensitization (Jin et al., 2015). These dyads comprise two BODIPY electron donor units attached to two adjacent carbon atoms of the central *o*-carborane, which serve as an acceptor group. Detailed synthetic routes for **BDP-*o*CB-12** and **BDP-*o*CB-13** are described in Scheme 8B. Through the reaction between the decaborane-Lewis base complex *arachno*-B_10_H_12_(SEt_2_)_2_ and alkyne derivatives **BDP-18** and **BDP-19**, it is possible to form the *o*-carborane moiety, resulting in the generation of **BDP-*o*CB-12** and **BDP-*o*CB-13**, respectively. The intermediate **BDP-18** and **BDP-19** were obtained following a literature procedure reported by Ziessel (Ulrich and Ziessel, 2004).

In both **BDP-*o*CB**-**12** and **BDP-*o*CB-13**, the electron-acceptor *o*-carborane group reduces the fluorescence intensities and lifetimes (Supplementary Table S2) of two electron-donor BODIPYs units through PET exergonic process, mainly from S_1_ to CT states. The calculated efficiencies were 63% and 71%, respectively. Theoretical calculations and cyclic voltammetry were also performed, supporting the PET hypothesis. Additionally, BODIPY^.+^ and CB^.-^ species generated through the PET process were identified via spectroelectrochemical (SEC) measurements.

Three years later, Kang and colleagues elucidated the dynamics of photoinduced electron transfer (PET) of **BDP-*o*CB-13** using femtosecond time-resolved transient absorption spectroscopy (Kim et al., 2018). The PET process occurs in the S1 state (Marcus normal region) and is strongly influenced by the driving force (-ΔG), which is controlled by solvent polarity; so, PET in the S_1_ state is faster in polar solvents than in nonpolar ones (τ_s1_ = 0.72 ns in CH_3_CN, 1.22 ns in CH_2_Cl_2_ and 2.72 ns in *n*-hexane). On the other hand, deactivation via internal conversion prevents the PET process occurring from the higher energy S_2_ state.

### 2.4 Fluorescent nanocars constructed from carborane-BODIPY systems

**SCHEME 9:**
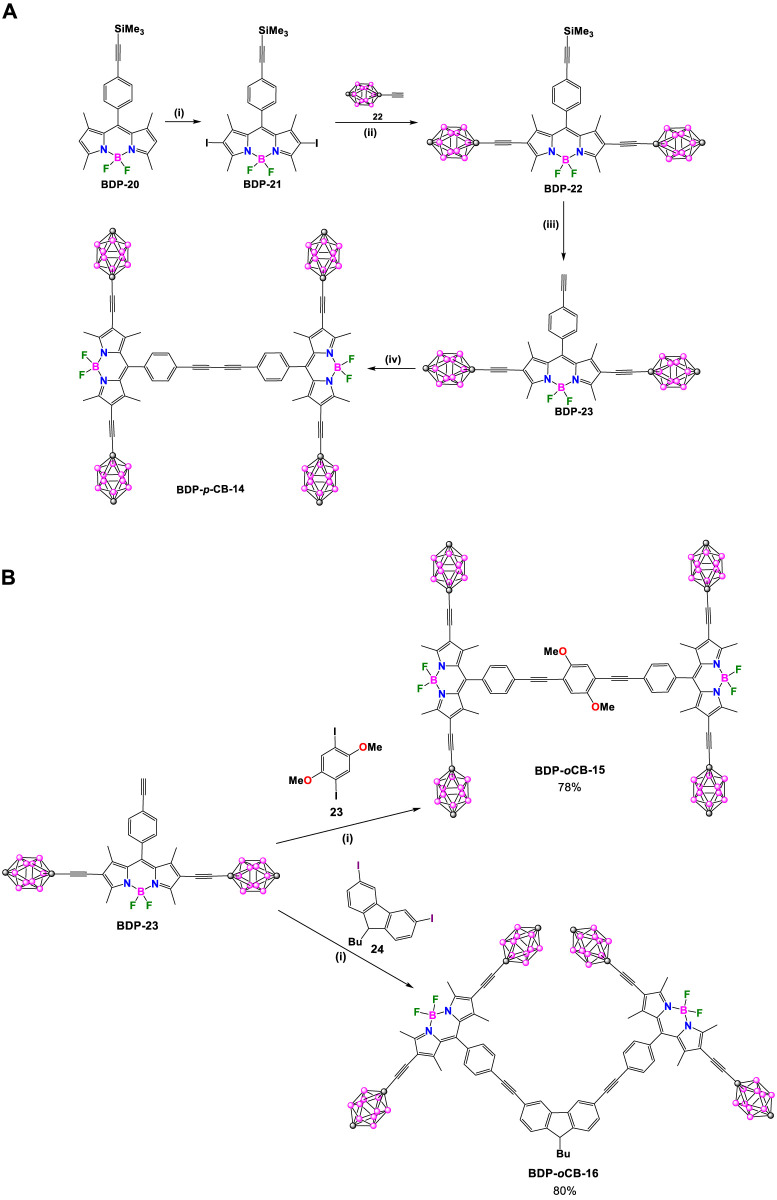
**(A)** Synthesis of **BDP-*p*CB**-**14**. Steps: (i) NIS, dimethylformamide (DMF), CH_2_Cl_2_, 84%; (ii) THF, Et_3_N, 60°C, PdCl_2_(PPh_3_)_2_, CuI, 88%; (iii) K_2_CO_3_, THF/MeOH, 79%; (iv) PdCl_2_(PPh_3_)_2_, CuI, air, THF, Et_3_N, 85%. **(B)** Synthesis of **BDP-*p*CB-15** and **BDP-*p*CB-16.** Reaction conditions: (i) PdCl_2_(PPh_3_)_2_, CuI, THF, Et_3_N, 50°C.

In recent decades, significant effort has been dedicated to designing, synthesizing, and operating synthetic molecular machines. Tour and coworkers have designed nanocars that can observe and track molecular motion using scanning tunneling microscopy (STM) and single-molecule fluorescence microscopy (SMFM), offering unparalleled atomic resolution (Morin et al., 2007; Khatua et al., 2009; Vives and Tour, 2009; Claytor et al., 2009). In 2010 they designed and synthesized three highly fluorescent nanocars: **BDP-*p*CB-14** (Scheme 9A), **BDP-*p*CB-15**, and **BDP-*p*CB-16** (Scheme 9B). (Godoy et al., 2010) The nanocar’s axle consists of two units of BODIPY, and four *p*-carborane units form part of the wheels, which are important in the translational motion. Nanocars **BDP-*p*CB-14** and **BDP-*p*CB-15** move in a straight line on surfaces. In contrast, nanocar **BDP-*p*CB-16** exhibits circular surface motion that could be detected by measuring the polarization anisotropy distribution. The compounds were prepared following a modular and convergent synthesis (Scheme 9). Axle **BDP-23** was obtained through a sequence of 4 steps with an overall yield of 58%. The first step involved a Sonogashira reaction between known **BDP-8** (Burghart et al., 1999) and ethynyltrimethylsilane resulting in compound **BDP-20**. Next, **BDP-20** underwent iodination at positions 2 and 6 using *N*-iodosuccinimide (NIS) to yield **BDP-21**. A double Sonogashira cross-coupling reaction between **BDP-22** and 1-ethynyl-*p*-carborane (**22**), followed by deprotection of the TMS group under basic conditions, allowed the formation of axle **BDP-23**. The homocoupling Eglinton-Glaser-type reaction, using PdCl_2_(PPh_3_)_2_ as a catalyst of **BDP-23**, provides nanocar **BDP-*p*CB-14**. For the synthesis of nanocars **BDP-*p*CB-15** and **16**, compound **BDP-23** was also used as an axle. Both compounds were synthesized by means of a double Sonogashira reaction using 1,4-diiodo-2,5-dimethoxybenzene (**23**) and 9-butyl-2,6-diiodo-9*H*-carbazole (**24**) as coupling patterns, respectively.

The optical properties of axle **BDP-23** and all nanocars were analyzed using UV-Vis and fluorescence spectroscopy, and the summarized data is provided in Supplementary Table S3. The three nanocars displayed intense absorption bands, and the values of extinction coefficients varied depending on the substitution pattern. The fluorescent QY of all nanocars was found to be slightly smaller than that of axle **BDP-23**, possibly due to through-bond energy transfer between the BODIPYs cores. The high fluorescent QYs make all the nanocars suitable for single-molecule fluorescence microscopy (SMFM) experiments.

### 2.5 AIE in carborane-BODIPY molecules for responsive materials and solid state devices

Some organic π-conjugated molecules exhibit aggregation-caused quenching (ACQ) (Jenekhe and Osaheni, 1994). Conversely, some organic π-conjugated molecules exhibit AIE, wherein non-emissive molecules in solution are induced to emit light under aggregate formation conditions; due to these characteristics, AIE-active compounds are top candidates for responsive materials and solid-state luminescent devices (Xie et al., 2024; Rana et al., 2023; Bhosle et al., 2024). It is well known that in solution, the *o*-carborane, acts as an electron-withdrawing group (Teixidor et al., 2000; Núñez et al., 2006), and triggers the PET process in the system, (Ferrer-Ugalde et al., 2014), when attaching to aromatic units through carborane C_c_ bonds. In addition, the phenyl units rotate around the C_c_-C bond. These two mechanisms, PET and rotation, increase the nonradiative decay, producing a quenching of the fluorescence emission. On the contrary, after aggregation in solution, the efficiency of PET and rotation decrease, and the phenomenon of AIE takes place, leading to an increase of the emission, confirming that the *o*-carborane acts as a good aggregation-induced emission generator (AIE-gen).

**SCHEME 10:**
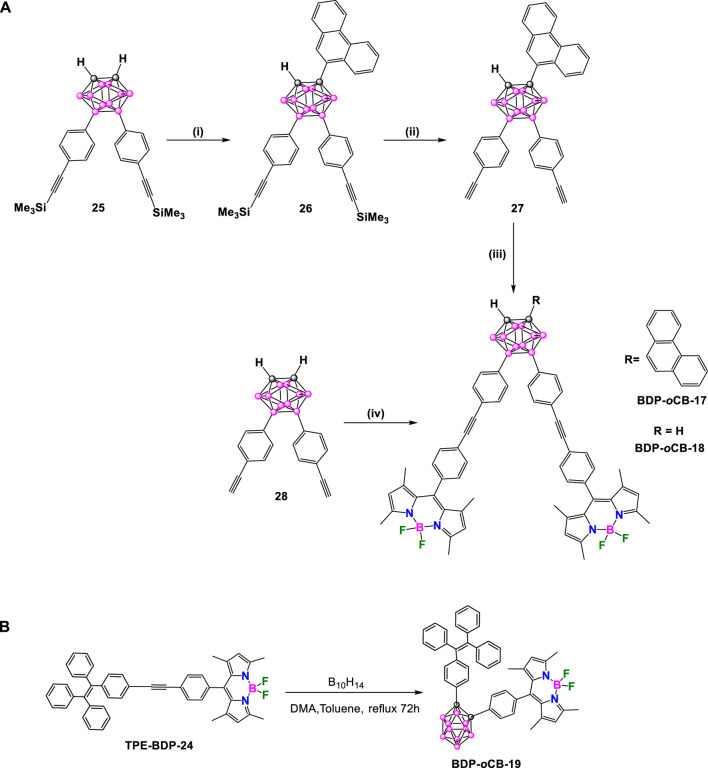
**(A)** Synthesis of **BDP-*o*CB-17** and **BDP-*o*CB-18**. Keys: (i) 1) *n*BuLi, MeOCH_2_CH_2_OMe, 0°C, 30 min, 2) CuCl, room temperature, 2 h, 3) 9-iodophenanthene, pyridine, reflux, 5 h, 27% for 3 steps; (ii) K_2_CO_3_, THF/MeOH, room temperature, 2 h, 93%; (iii) **BDP-8**, Pd(PPh_3_)_4_, CuI, THF/Et_3_N, 40°C, 15 h, 33%; (iv) **BDP-8**, Pd(PPh_3_)_4_, CuI, THF/Et_3_N, 40°C, 15 h, 33%. **(B)** Synthesis of **BDP-*o*CB-19**.

In 2014, Chujo and coworkers synthesized a series of organic dyes based on *o*-carborane with the aim of studying emission behaviors, intramolecular charge transfer (ICT), energy transfer, AIE, and ACQ (Tominaga et al., 2014). Two organic dyes, **BDP-*o*-CB-17** and **BDP-*o*CB-18** (Scheme 10A), contain tolane-BODIPY units linked to the 9,12-boron position of *o*-carborane were prepared. Additionally, **BDP-*o*CB-17** has a phenanthrene unit linked to the C_c_ position of *o*-carborane. Compound **25** (Tominaga et al., 2013) was treated with *n*BuLi to deprotonate the C_c_. Subsequent Ullman-type coupling with 9-iodophenanthrene formed compound **26**. The removal of trimethylsilyl (TMS) groups of **26** using K_2_CO_3_ as a base provided compound **27**. Finally, a Sonogashira cross-coupling reaction between **27** and **BDP-8** afforded the target molecule **BDP-*o*CB-17**. On the other hand, the **BDP-*o*CB-18** was synthesized via a Sonogashira coupling between compound **28** and **BDP-8**.

Supplementary Table S4 outlines the results from the absorption and photoluminescence spectroscopy analyses. **BDP-*o*CB-17** and **BDP-*o*CB-18** have similar emission behaviors. In a THF dilute solution, both exhibited intense photoluminescence upon excitation at 310 nm, emitting green light from the BODIPY moiety. **BDP-*o*CB-17** and **BDP-*o*CB-18** have fluorescence QYs of 0.33 and 0.40, respectively. In contrast, neither dye emits in both aggregate and solid state due to energy transfer from tolanes and phenanthrene (in **BDP-*o*CB-17**) to the BODIPY cores, and the subsequent ACQ mechanism.

In 2019, Hamuryudan et al. synthesized the BODIPY triad **BDP-*o*CB-19** (Nar et al., 2019) following a similar process to one previously reported by the same authors (Berksun et al., 2018). The decaborane insertion to the ethynyl derivative TPE-BDP-24 in dry toluene gave the BODIPY triad BDP-oCB-19 in a 47% yield (Scheme 10B).

**BDP-*o*CB-19** displayed two absorption bands in THF, the one at 311 nm was attributed to an electronic transition located at the tetraphenylethylene (TPE), whereas the band at 502 nm was associated with BODIPY absorption (Nar et al., 2019). The absorption spectrum was similar to their precursors, TPE and BODIPY, suggesting that there is not considerable ground-state interaction between the fluorophores through the *o*-carborane. Furthermore, the emission properties of the compound in mixtures of THF/water indicated that the emission intensity increased by the increasing upon adding water to THF due to the formation of aggregates, what suggested that **BDP-*o*CB-19** exhibits AIE properties in THF/water solutions. **BDP-*o*CB-19** did not show AIE properties in the mixture of solvents, but an aggregation-caused quenching (ACQ) was observed with a subsequent decrease in the emission. The observed ACQ effect was explained by structural considerations (*vide infra*). In one hand, methyl groups on the BODIPY unit suppress an intramolecular charge transfer (ICT) between BODIPY and TPE, due to the electron donating character of the methyl groups to the BODIPY moiety. On the other hand, crystal packing shows face-to-face π–π stacking of BODIPY units with a short distance of 3.97 Å between BODIPY–BODIPY mean planes for both compounds, which could explain the formation of aggregates that lead to ACQ behavior.

## 3 BODIPY dyes enhanced with boron clusters for biological applications

Carborane-BODIPY dyes have emerged as promising candidates for biological applications due to their unique photophysical properties and biocompatibility. These dyes combine the strong fluorescence and stability of BODIPY compounds with the advantageous features of carborane clusters, such as increased lipophilicity and enhanced cellular uptake. Recent studies have demonstrated their effectiveness as fluorescent markers in imaging techniques, enabling visualization of biological processes at the cellular level. Additionally, the incorporation of boron cluster structures can improve the dyes’ stability and resistance to photobleaching, making them suitable for long-term observations. As a result, carborane-BODIPY dyes are being explored for use in diagnostic tools, targeted drug delivery, and real-time tracking of biological events.

### 3.1 BODIPY and aza-BODIPY dyes functionalized with *o* and *m*-carborane derivatives

**SCHEME 11:**
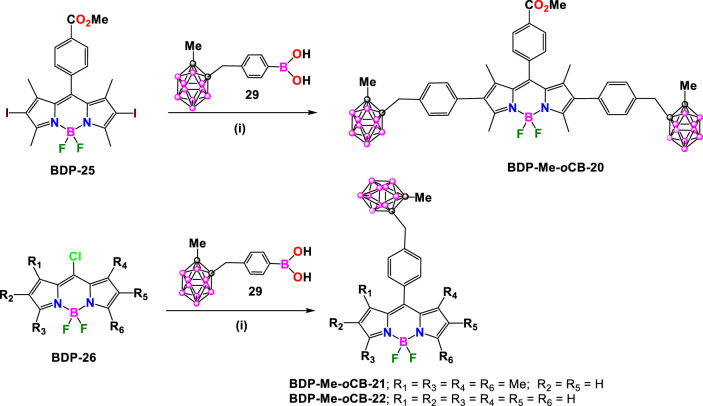
Synthesis of **BDP-Me-*o*CB-20**, **BDP-Me-*o*CB-21**, **BDP-Me-*o*CB-22.** Reactions conditions: (i) Na_2_CO_3_, Pd(PPh_3_)_4_, toluene.

In 2015, M.G. Vicente et al. synthesized the BODIPY **BDP-Me-oCB-20** by Suzuki coupling reaction between the diiodo BODIPY **BDP-25** with 1.5 equiv. of *o*-carborane-containing boronic acid (**29**) (Hao et al., 2007), in the presence of Na_2_CO_3_ and Pd(PPh_3_)_4_ (Gibbs et al., 2015). The reaction afforded **BDP-Me-*o*CB-20** in 89% yield after purification (Scheme 11). In parallel, the reaction of **29** with the corresponding 8-chloro-BODIPY **BDP-26** under the same conditions led to the formation of **BCP-Me-*o*CB-21** (Gibbs et al., 2015) and **BCP-Me-*o*CB-22** (Xuan et al., 2016) in 47% and 45% yield, respectively (Scheme 11).

**SCHEME 12:**
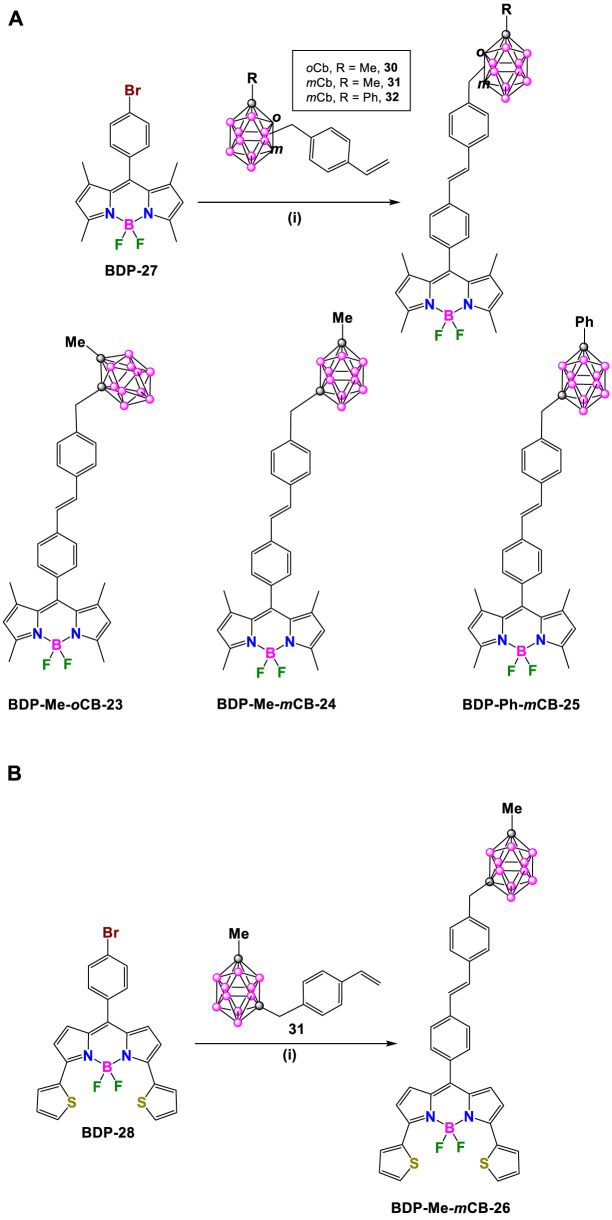
**(A)** Synthesis of **BDP-Me-*o*CB-23, BDP-Me-*m*CB-24** and **BDP-Ph-*m*CB-25**. Reaction conditions: (i) [Pd_2_ (dba)_3_] (1.2 mol%), Pd [P (*t*-Bu)_3_]_2_ (1.6 mol%), Cy_2_ *N*Me (1.4 eq.), dry 1,4-dioxane, 100°C, overnight. **(B)** Synthesis of **BDP-Me-*m*CB-26**. Reaction conditions: (i) [Pd_2_ (dba)_3_] (1.2 mol%), Pd [P (*t*-Bu)_3_]_2_ (1.6 mol%), Cy_2_ *N*Me (1.4 eq.), dry 1,4-dioxane, 100°C, 12 h.

Later in 2018, R. Núñez, C. Prandi and coworkers synthesized a family of carborane-BODIPY dyes (Bellomo et al., 2018) by Heck cross-coupling reaction between the green emitting brominated **BDP-27** (Gibbs et al., 2013) and styrene-substituted Me-*o*-carborane (**30)**, Me-*m*-carborane (**31**) or Ph-*m*-carborane (**32**) (Ferrer-Ugalde et al., 2012), in the presence of Pd_2_ (dba)_3_/Pd(P(tBu)_3_)_2_ (1.2 and 1.6 mol%, respectively) as catalysts, and Cy_2_ *N*Me as a base (Littke and Fu, 2001) in 1,4-dioxane at 100°C for 12 h. After an appropriate purification process, the formation of the expected carboraryl-BODIPY dyes **BDP-Me-*o*CB-23**, **BDP-Me-*m*CB-24** and **BDP-Ph-*m*CB-25** in moderate to good yields (46%, 51%, and 67%, respectively) (Scheme 12A) took place. In addition, the reaction of **BDP-28** and **31** under the same conditions yielded **BDP-Me-*m*CB-26** with a 55% yield (Scheme 12B).

**SCHEME 13:**
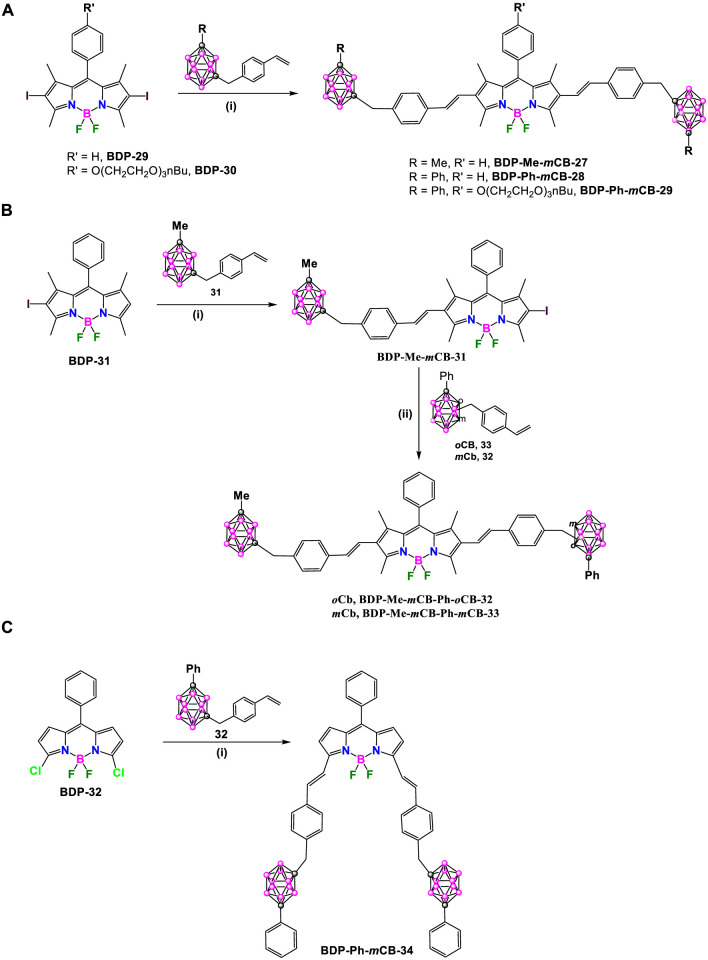
**(A)** Synthesis of carborane-BODIPY dyes **BDP-Me-*m*CB-27**, **BDP-Ph-*m*CB-28** and **BDP-Ph-*m*CB-29**. Reaction conditions: (i) **styrenyl*-CB:* 31** or **32** (1 eq.), [Pd_2_(dba)_3_] (3 mol%), Pd[P(*t*-Bu)_3_]_2_ (6 mol%), Cy_2_ *N*Me (5 eq.), dry 1,4-dioxane, 100°C, 12 h dry 1,4-dioxane, 100°C, overnight. **(B)** Synthesis of carborane-BODIPY dyes **BDP-Me-*m*CB-Ph-*o*CB-32** and **BDP-Me-*m*CB-Ph-*m*CB-33**. Reaction conditions: (i) 1) [Pd_2_(dba)_3_] (1.2 mol%), Pd[P(*t*-Bu)_3_]_2_ (1.6 mol%), Cy_2_ *N*Me (1.4 eq.), dry 1,4-dioxane, 100°C, overnight, 2) *N*-iodosuccinimide (2 eq.), DCM, r.t, 12 h (ii) [Pd_2_(dba)_3_] (1.2 mol%), Pd[P(*t*-Bu)_3_]_2_ (1.6 mol%), Cy_2_ *N*Me (1.4 eq.), dry 1,4-dioxane, 100°C, overnight. **(C)** Synthesis of **BDP-Ph-*m*CB-34**. Reaction conditions: (i) [Pd_2_(dba)_3_] (3 mol%), Pd[P(*t*-Bu)_3_]_2_ (6 mol%), Cy_2_ *N*Me (4.8 eq.), dry 1,4-dioxane, 100°C, overnight.

Later, in 2021, the same group synthesized a set of carborane-BODIPY conjugates (Bellomo et al., 2021). The Heck coupling reaction between the 2,6-diiodo BODIPY **BDP-29** and two equivalents of **31** in refluxing 1,4-dioxane using the [Pd_2_ (dba)_3_] and [Pd (tBu_3_P)_2_] as catalysts, in the presence of Cy_2_ *N*Me overnight at reflux, yielded **BDP-Me-*m*CB-27** in 43% yield, along with its β,β-isomer (Scheme 13A). (Bellomo et al., 2021). The same conditions were successfully applied to the reaction of **BDP-29** or **BDP-30**, which incorporate an amphiphile oligoethylene glycol alkyl chain at the *meso*-position, with the styrenyl substituted *m*-carborane derivative **32** to achieve symmetric dyes **BDP-Ph-*m*CB-28** and **BDP-Ph-*m*CB-29**, in 57% and 52%, respectively (Scheme 13A).

Once the reaction was optimized, the procedure was extended to the synthesis of asymmetric compounds by attaching two different carboranyl units to the starting BODIPY. The mono-iodinated 1,3,5,7-tetramethyl BODIPY, **BDP-31** was used as starting compound, that reacted in a first step with **31** in the presence of [Pd_2_ (dba)_3_], [Pd (tBu_3_P)_2_] and Cy_2_ *N*Me (1.4 eq.) in refluxing dry 1,4-dioxane to give intermediate **BDP-Me-*m*CB-30** in 72% (Scheme 13B). The reaction of **BDP-Me-*m*CB-30** with *N*-iodosuccinimide at room temperature overnight leads to the formation of compounds **BDP-Me-*m*CB-31**, that after the reactions with **33** and **32** under Heck conditions gave asymmetric dyes **BDP-Me-*m*CB-Ph-*o*CB-32** and **BDP-Me-*m*CB-Ph-*m*CB-33** in 55% and 35%, respectively (Scheme 13B). Following similar conditions as before, **BDP-Ph-*m*CB-34** was synthesized by the Heck-coupling reaction between **BDP-32** and styrenyl-carborane **32** (Scheme 13C).

**SCHEME 14:**
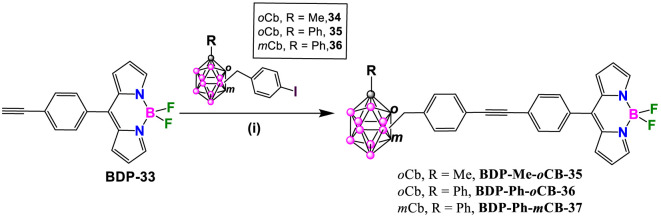
Synthesis of **BDP-Me-*o*CB-35**, **BDP-Ph-*o*CB-36** and **BDP-Ph-*m*CB-37**. Reaction conditions: (i) carboranyl benzyl iodide (1 mmol), Pd(PPh_3_)_2_Cl_2_ (0.05 mmol), CuI (0.1 mmol), THF, Et_3_N, 2h reflux.

BODIPY dye **BDP-33** reacted with carborane derivatives **34**, **35** and **36** (Ferrer-Ugalde et al., 2017), under Sonogashira cross-coupling reaction conditions, by using Pd(PPh_3_)_2_Cl_2_ and CuI, in a solution of THF and triethylamine at reflux for 2 h (Scheme 14). After that time, the solvent was evaporated and the residue was dissolved in DCM and washed with saturated ammonium chloride. Finally, compounds were purified through a silica gel chromatographic column using hexane/acetone mixtures as eluent to give **BDP-Me-*o*CB-35**, **BDP-Ph-*o*CB-36** and **BDP-Ph-*m*CB-37** in 56, 81, 61% yield, respectively (Labra-Vázquez et al., 2020).

**SCHEME 15:**
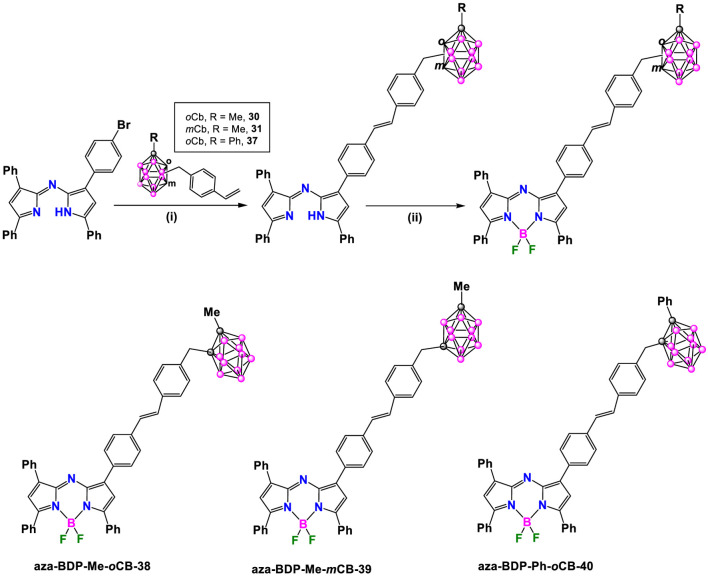
Synthesis of carborane-aza-BODIPY dyes **aza-BDP-Me-*o*CB-38**, **aza-BDP-Me-*m*CB-39** and **aza-BDP-Ph-*o*CB-40**. Reaction conditions: (i) [Pd_2_ (dba)_3_] (1.2 mol%), Pd [P (*t*-Bu)_3_]_2_ (1.6 mol%), Cy_2_ *N*Me (1.4 eq.), dry 1,4-dioxane, 100°C, 12 h; (ii) BF_3_·OEt_2_, DIPEA, DCM.

Furthermore, the synthesis of red/NIR emitting carboranyl-aza-BODIPY derivatives was also performed by Bellomo et al. (Bellomo et al., 2018), following the Heck coupling reaction; however, due to the tendency of the aza-BODIPY to dechelate under the cross-coupling conditions (Parisotto et al., 2017; Bellier et al., 2011), this reaction was initiated using the brominated 3,3′,5,5′-tetraarylazadipyrromethene dye **aza-DIPY** that reacted with **30**, **31** and **37** (Scheme 15), in the presence of Pd_2_ (dba)_3_/Pd(P(tBu)_3_)_2_, Cy_2_Me in 1,4-dioxane at reflux overnight to give the corresponding carboranyl-substituted aza-DIPY **aza-DP-Me-*o*CB-1, aza-DP-Me-*m*CB-2** and **aza-DP-Ph-*o*CB-3**. The last step was the reaction with BF_3_·OEt_2_ using diisopropylethylamine (DIPEA) in DCM to give compounds **aza-BDP-Me-*o*CB-38**, **aza-BDP-Me-*m*CB-39** and **aza-BDP-Ph-*o*CB-40** in 93%, 88%, and 86% yields, respectively) (Scheme 15).

All these carborane-BODIPY and aza-BODIPY derivatives exhibited characteristic absorption spectra of BODIPY dyes with strong absorption bands corresponding to the S_0_-S_1_ (π-π*) transitions. These bands are accompanied by a shoulder at slightly lower wavelength attributed to vibrational transitions. The maximum absorption (λ_max_) for these compounds ranged from 501 to 641 nm (Supplementary Table S5). For the same set of compounds, similar absorption spectra were obtained and no significant effect of the different carboranes were observed. Notably, BODIPY **BDP-Me-*o*CB-34** (Scheme 13C) showed the largest red-shift (up to 140 nm) compared to the others BODIPYs, attributed to the substitution at the 3,5 or α positions, followed by those BODIPYs with 2,6-aryl substitution (Jiao et al., 2011). Those BODIPYs substituted with carboranes at the *meso* position exhibited a hypsochromic shift in the absorption maxima (Supplementary Table S6). The same trend was observed in the emission spectra with λ_em_ ranging from 508 nm for **BDP-Me-oCB-21** (Scheme 11) to 641 nm for **BDP-Me-oCB-34** (Scheme 13C). In the case of **BDP-Me-*m*CB-26** (Scheme 12B), as expected, a significant red-shift was observed when compared with its homologous, **23**, **24** and **25** (Scheme 12A), due to the conjugation of the thiophene group in 3 and 5 positions. The large ranges of absorption and emission is reflected in the different Stokes shifts observed from 7 to 65 nm (Supplementary Table S5). Regarding the fluorescence QYs, **BDP-Me-*o*CB-20** and **BDP-Me-*m*CB-21** (Scheme 11) exhibited the highest values of around 99% (Gibbs et al., 2015), which indicated that the *o*-carborane cluster not only did not quench the fluorescence but also enhanced the luminescence properties of the fluorophores. (Ferrer-Ugalde et al., 2012; Ferrer-Ugalde et al., 2014). On the contrary, the decrease of fluorescence QY observed in **BDP-Me-*o*CB-22** (Scheme 11) was attributed to a higher rotational freedom of the 8-aryl group in the absence of methyl groups. **BDP-Me-*o*CB-23**, **BDP-Me-*m*CB-24**, and **BDP-Ph-*m*CB-25** (Scheme 12A) showed good to moderate QYs, ranging from 0.40 to 0.44, which was attributed to the restricted rotation of the *meso*-aryl group (Karatay et al., 2015). However, the rest of BODIPYs exhibited low quantum efficiencies, specially **BDP-Me-*o*CB-35**, **BDP-Ph-*o*CB-36** and **BDP-Ph-*m*CB-37** (Scheme 14) with QYs ∼ 0.01. The solvatochromic analysis of **BDP-Me-*m*CB-27**, **BDP-Ph-*m*CB-28** (Scheme 13A), **BDP-Me-mCB-Ph-*o*CB-32** (Scheme 13B) and **BDP-Ph-*m*CB-34** (Scheme 13C) indicated that a very minor solvent effect was observed both in absorption and emission maxima for the four compounds due to their low intrinsic molecular dipole moment. Aggregation studies in water for **BDP-Ph-*m*CB-28** and **BDP-Ph-*m*CB-29** (Scheme 13A) showed a similar photophysical behavior upon increasing amount of water. Up to 40% of water in THF, a hypochromic effect in the absorption spectra occurred, whereas similar emission spectra to those exhibited in pure THF were observed. In those THF:water mixtures (60/80 v/v), the absorption spectra showed large, red-shifted peaks suggesting the formation of aggregates, and the emission bands were weak most likely due to aggregation phenomena. A clear absorption band was observed in water indicating the formation of stable aggregates, making these BODIPYs excellent candidates as fluorogenic probes for bio-supramolecular assays, in which the fluorescence features might get restored upon binding or intercalation events with biological relevant molecules. For the aza-BODIPYs, both absorption and emission maxima were characteristics for these kind of dyes with maxima centered at 665 and 706 nm, respectively, exhibiting a noticeable bathochromic shift when compared to their parent tetraphenyl-BODIPYs and very low fluorescence quantum yields (Supplementary Table S5).

### 3.2 BODIPY dyes functionalized with mercaptocarborane derivatives

**SCHEME 16:**
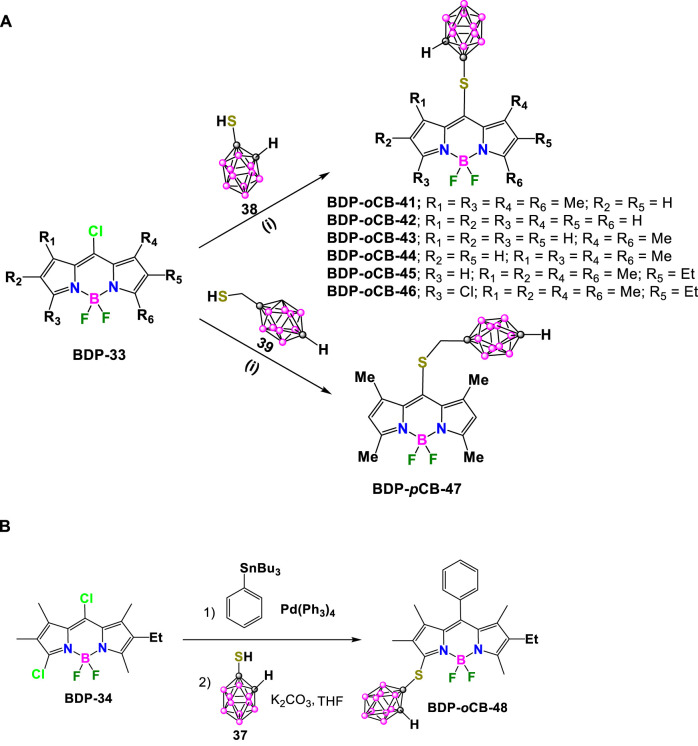
**(A)** Synthesis of **BDP-*o*CB-41**, **BDP-*o*CB-42**, **BDP-*o*CB-43**, **BDP-*o*CB-44**, **BDP-*o*CB-45**, **BDP-*o*CB-46** and **BDP-*p*CB-47.** Reaction conditions: (i) K_2_CO_3_, THF, r.t. **(B)** Synthesis of **BCP-*o*CB-48**.

In 2015, M. G. Vicente reported the synthesis of a family of mercaptocarborane-BODIPY dyes as boron delivery agents for their potential application in BNCT (*vide infra*). The substitution reaction of the corresponding 8-chloro-BODIPY **BDP-33** with 1-mercapto-*o*-carborane (**38**) in the presence of K_2_CO_3_ in THF to obtain **BDP-*o*CB-41** in 94% yield (Scheme 16A). (Gibbs et al., 2015) Later, the same synthetic methodology was used to prepare the family of compounds **BDP-oCB-42 - BDP-oCB-46**, with yields ranging from 74% to 95% (Xuan et al., 2016). Following the same procedure and conditions, the reaction of the **BDP-30** with 1-mercaptomethyl-*p*-carborane (**39**) led to **BDP-pCB-47** in 92% yield. (Wang et al., 2014) (Scheme 16A). On the other hand, **BDP-*p*CB-48** was prepared in 67% yield from **BDP-34** via two successive regioselective reactions, a Stille cross-coupling at the 8-position using 1 equiv of tributylphenylstannane and Pd(PPh_3_)_4_, followed by substitution using an excess of **37** (Scheme 16B). (Zhao et al., 2015)

**SCHEME 17:**
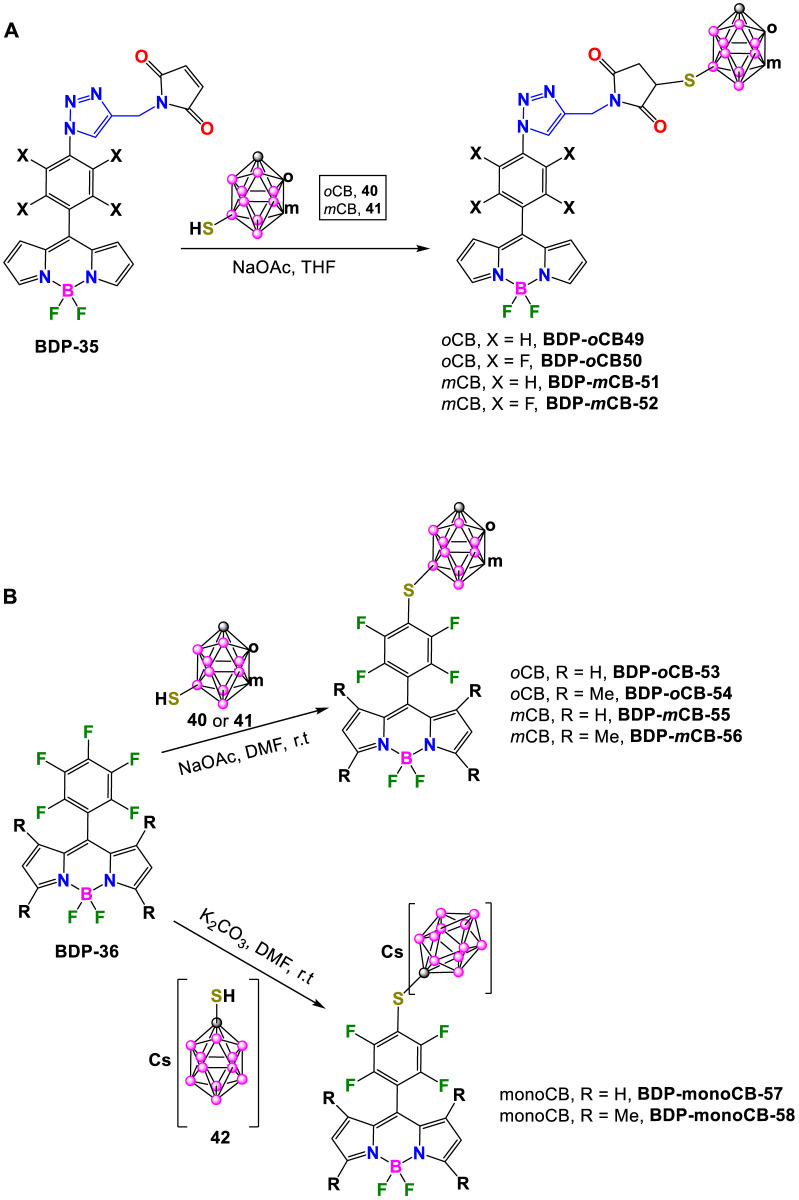
**(A)** Synthesis of **BDP-*o*CB-49**, **BDP-*o*CB-50**, **BDP-*m*CB-51** and **BDP-*m*CB-52. (B)** Synthesis of **BDP-*o*CB-53**, **BDP-*o*CB-54**, **BDP-*m*CB-55**, **BDP-*m*CB-56**, **BDP-monoCB-57** and **BDP-monoCB-58**.

Later in 2022, Ol’shevskaya and coworkers reported another set of mercaptocarborane-BODIPY conjugates for biological applications (*vide infra*). The reaction of **BDP-35** that contain triazole linkers modified with maleimides, with the 9-mercapto-*o*-carborane (**40**) or 9-mercapto-*m*-carborane (**41**) (Plesek et al., 1978) takes place at the maleimide double bonds in the presence of NaOAc in THF, to produce thiosuccinimide compounds **BDP-*o*CB-49**, **BDP-*o*CB-50**, **BDP-*m*CB-51** and **BDP-*m*CB-52** in 40%–75% yields as red crystalline solids (Scheme 17A). (Zaitsev et al., 2022b)

The same authors have demonstrated that the thiolation reaction of pentafluorophenyl groups in **BDP-36** (Alamiry et al., 2012) with mercaptocarboranes **40** or **41** in the presence of NaOAc in DMF for 1 h at r.t. produced the corresponding carborane-substituted BODIPYs **BDP-*o*CB-53**, **BDP-*o*CB-54**, **BDP-*m*CB-55** and **BDP-*m*CB-56** in 60%–68% yields (Scheme 17B). (Zaitsev et al., 2022a) Besides, two water soluble boronated BODIPYs **BDP-monoCB-57** and **BDP-monoCB-58** were also prepared in 72% and 83% yields respectively, by reaction of **BDP-36** and the anionic 1-mercapto-1-carba-*closo*-dodecaborate (**42**) (Jelínek et al., 1986) using K_2_CO_3_ in anhydrous DMF at room temperature for 8 h (Scheme 17B).

**SCHEME 18:**
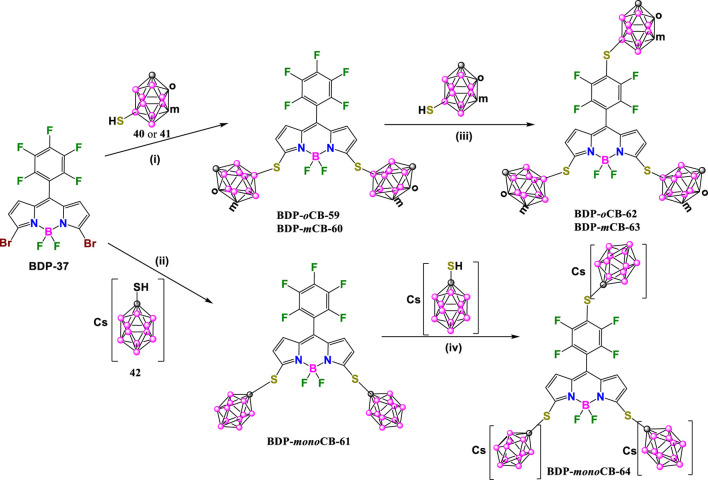
Synthesis of **BDP-*o*CB-62**, **BDP-*m*CB-63** and **BDP-monoCB-64**. Reaction conditions (i) NaOAc, CH_3_CN, reflux 2h; (ii) K_2_CO_3_, CH_3_CN, reflux 4h; (iii) NaOAc, DMF, r.t 1 h; (iv) K_2_CO_3_, DMF, r.t. 4 h.

Following a similar procedure with slight modifications, A. Ol’shevskaya and coworkers have recently prepared a new family of carborane-BODIPY derivatives (Zaitsev et al., 2023). The reaction of **BDP-37** (Sekhar et al., 2014) with mercaptocarboranes **40** or **41** in the presence of NaOAc in boiling CH_3_CN under argon for 2 h afforded **BDP-*o*CB-59**, **BDP-*m*CB-60** in 85% and 92% yields, respectively (Scheme 18). A similar reaction of **BDP-37** with water-soluble **42** in the presence of K_2_CO_3_ in CH_3_CN at reflux for 4 h led to the formation of **BDP-monoCB-61** in 70% yield (Scheme 18). It is important to note that these reaction conditions did not produce *p*-fluorine substitution, so it is necessary to modify the conditions to introduce the carborane clusters at the pentafluorophenyl group in the new BODIPYs. The reaction of **BDP-*o*CB-59** or **BDP-*m*CB-60** with the corresponding mercapta-carboranes in the presence of NaOAc in DMF at room temperature for 1 h gave **BDP-*o*CB-62** and **BDP-*m*CB-63** in 80% and 75% yields, respectively. In the same way, compound **BDP-monoCB-64** was obtained in 71% yield by reaction of **BDP-monoCB-61** with **42** and K_2_CO_3_ in DMF at room temperature for 4 h (Scheme 18).

Dyes **BDP-*o*CB-41–48** (Scheme 16) displayed characteristic absorption spectra of BODIPYs with maxima in the range 521–582 nm (Supplementary Table S6). The introduction of the *o*-carboranylthio group at the *meso* (8) position (Wang et al., 2014) caused a significant red-shift (up to 57 nm) in the maximum absorption compared to analogs **BDP-*oCB*-21** and **BDP-*o*CB-22** (Scheme 11). Regarding emission properties, these BODIPYs exhibited emission maxima ranging from 540 to 609 nm. As observed in their absorption properties, BODIPYs **BDP-*o*CB-45** and **BDP-*o*CB-46** (Scheme 16A) showed a significant red-shift in emission (up to 61 nm) compared to the others BODIPYs (Supplementary Table S6). This red shift is likely due to the stabilization of the LUMO by the *o*-carboranylthio group, which decreases the HOMO–LUMO gap. It is well known that the replacement of the *meso*-chloro by *me*so-thiol produces an important decrease in the fluorescence. While **BDP-*o*CB-41** and **BDP-*o*CB-44** to **BDP-*o*CB-47** are poorly emissive, (Wang et al., 2014), **BDP-*o*CB-42** showed higher fluorescence quantum yield, suggesting limited rotational freedom for the 8-carboranylthio group (Supplementary Table S6). The yields decrease with increasing alkyl substitution in **BDP-*o*CB-43** to **BDP-oCB-45**, due to increased energy loss to nonradiative deactivation processes.

The absorption spectra of **BDP-*o*CB-53**, **BDP-*o*CB-54**, **BDP-*m*CB-55**, **BDP-*m*CB-56**, **BDP-monoCB-57** and **BDP-monoCB-58** (Scheme 17B) in EtOH exhibited very similar characteristics. The absorption maxima for these compounds were in the range 513–515 nm with a noticeable shoulder in the region 480–490 nm. In the same solvent, these compounds displayed fluorescence spectra with emission maxima in the range 528–538 nm. They also exhibited very high fluorescence QYs ranging from 62% to 100% (Supplementary Table S7). The results indicated that methyl-substituted BODIPY derivatives exhibited higher fluorescence efficiency compare to un-substituted derivatives. This is attributed to the restricted rotation of the *meso-*tetrafluorophenyl moiety around the BODIPY core in the methyl-substituted BODIPYs, which decreases the internal conversion rate. In contrast, the *meso*-tetrafluorophenyl group in unsubstituted derivatives rotates more freely, leading to increased non-radiative decay processes. Additionally, the presence of the carborane clusters did not affect the non-radiative decay pathway.

Compounds **BDP-*o*CB-59**, **BDP-*m*CB-60** and **BDP-monoCB-61** (Scheme 18), where the carboranyl substitution was at 3 and 6 positions of the BODIPY core instead of the pentafluorophenyl group, showed a red-shift of the absorption maxima in acetone solutions compared to previous dyes (Supplementary Table S7). **BDP-*o*CB-59** and **BDP-*m*CB-60** showed absorption maxima at 596 and 593 nm respectively, whereas dianionic **BDP-monoCB-61** displayed a maximum at 607 nm. Similar absorption maxima are observed for **BDP-*o*CB-62**, **BDP-*m*CB-63**, and **BDP-monoCB-64** (Scheme 18), with molar extinction coefficients ranging from 75,500 to 85,600 M^−1^cm^−1^. All compounds in acetone showed similar emission spectra, with maxima in the range 612–632 nm, red-shifted compared to previous dyes, where the carborane cluster is attached to the pentafluophenyl group. Notably, **BDP-monoCB-61** and **BDP-monoCB-64** exhibited the longest emission maxima at 625 and 632 nm respectively. This suggests that anionic mono-carborane clusters cause a more significant bathochromic shift. Additionally, these compounds have lower fluorescence QYs of 89% and 91% (64% in water) compared to the neutral derivatives. Notably, linking carborane clusters through sulfur to the BODIPY core results in a red-shift in both absorption and emission spectra compared to compounds where the carborane is linked to the pentafluorophenyl group. In addition, the anionic monocarborane clusters produce larger red-shift than the neutral carborane, and lower quantum efficiencies.

### 3.3 X-ray structures analysis of boron clusters substituted BODIPYs

The spectral properties of BODIPY dyes can be easily modulated due to the manipulability of their structural backbone. Historically, modifications at the 3,5- and 2,6-positions of BODIPY-based probes (Chart 1) have resulted in red-shifted emission wavelengths and enhanced water solubility (Loudet and Burgess, 2007). Building on these findings, recent research into *meso*-substituents (i.e., 8-position substituents) has significantly impacted spectral properties, including fluorescence wavelengths, quantum yields, and Stokes shifts (Yan et al., 2022). For example, the relative rotation of the meso-aryl groups to the BODIPY core plane can greatly affect the excited-state dynamics of the dyes. Thus, an increase of QY is usually observed when such rotation is restricted (Kee et al., 2005). The substituents nature introduced into the various positions of the BODIPYs are very diverse and have been previously reviewed. In this review section We have only considered the reported X-ray crystal structures of carborane-functionalized BODIPYs designed for biological applications. We have compared the molecular structures and conformations within each family of compounds and related them to the photophysical properties.

#### 3.3.1 Mercaptocarboranyl-meso-BODIPYs

**FIGURE 1:**
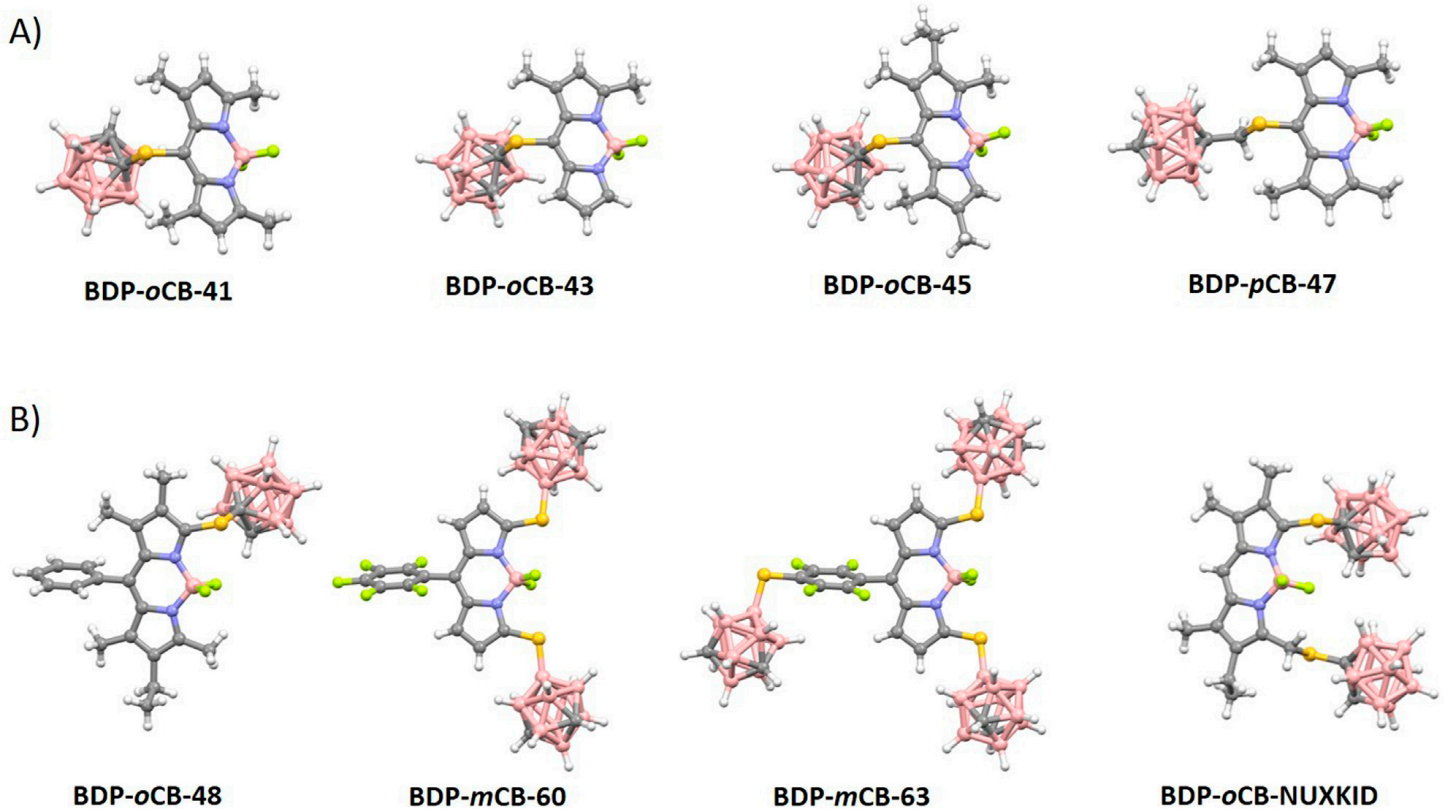
**(A)** Molecular structures for *meso*-substituted carboranylthio BODIPYs (Gibbs et al., 2015; Xuan et al., 2016; Wang et al., 2014). Color code: B, pink; C, grey; H, white; S, yellow; N, blue; and F, green. **(B)** Molecular structures for 3/5-substituted carboranylthio BODIPYs (Zaitsev et al., 2023; Zhao et al., 2015). Color code: B, pink; C, grey; H, white; S, yellow; N, blue; and F, green.

Structural analysis of all carboranylthio-*meso*-BODIPYs (Figure 1A) confirmed a restricted rotation through the C-S bond of the carboranylthio groups relative to the BODIPY rings (Supplementary Table S8). Torsion angles between 86° and 100° indicate that the carboranylthio groups are essentially perpendicular to the BODIPY planes. The presence of methyl groups at positions 1 and/or 7 of the BODIPY rings (Chart 1A; Figure 1A) appears to hinder this rotation. As previously discussed QYs increase with increased alkyl substitution on the BODIPY ring due to the reduced rotational freedom of the carboranylthio group. Nevertheless, the introduction of the *meso*-thiol provokes an important decrease of the fluorescence efficiency (Supplementary Table S8).

#### 3.3.2 Mercaptocarboranyl-pyrrole-BODIPYs

In contrast to the carboranylthio-*meso*-BODIPYs, the molecular structure of the corresponding derivatives with substitution at positions 3- and/or 5- of the BODIPY moiety reveals that the carboranylthio groups can freely rotate relative to the BODIPY plane (Figure 1B; Supplementary Table S9). The rotation angles in these structures range from 9° (nearly coplanar) to 90° (perpendicular). It is noticeable that the substitution of the thiolcarborane at these positions produces a significant increase of the QYs, that even reach 100% (Supplementary Table S9).

#### 3.3.3 Carboranyl-aryl-meso-BODIPYs

**FIGURE 2:**
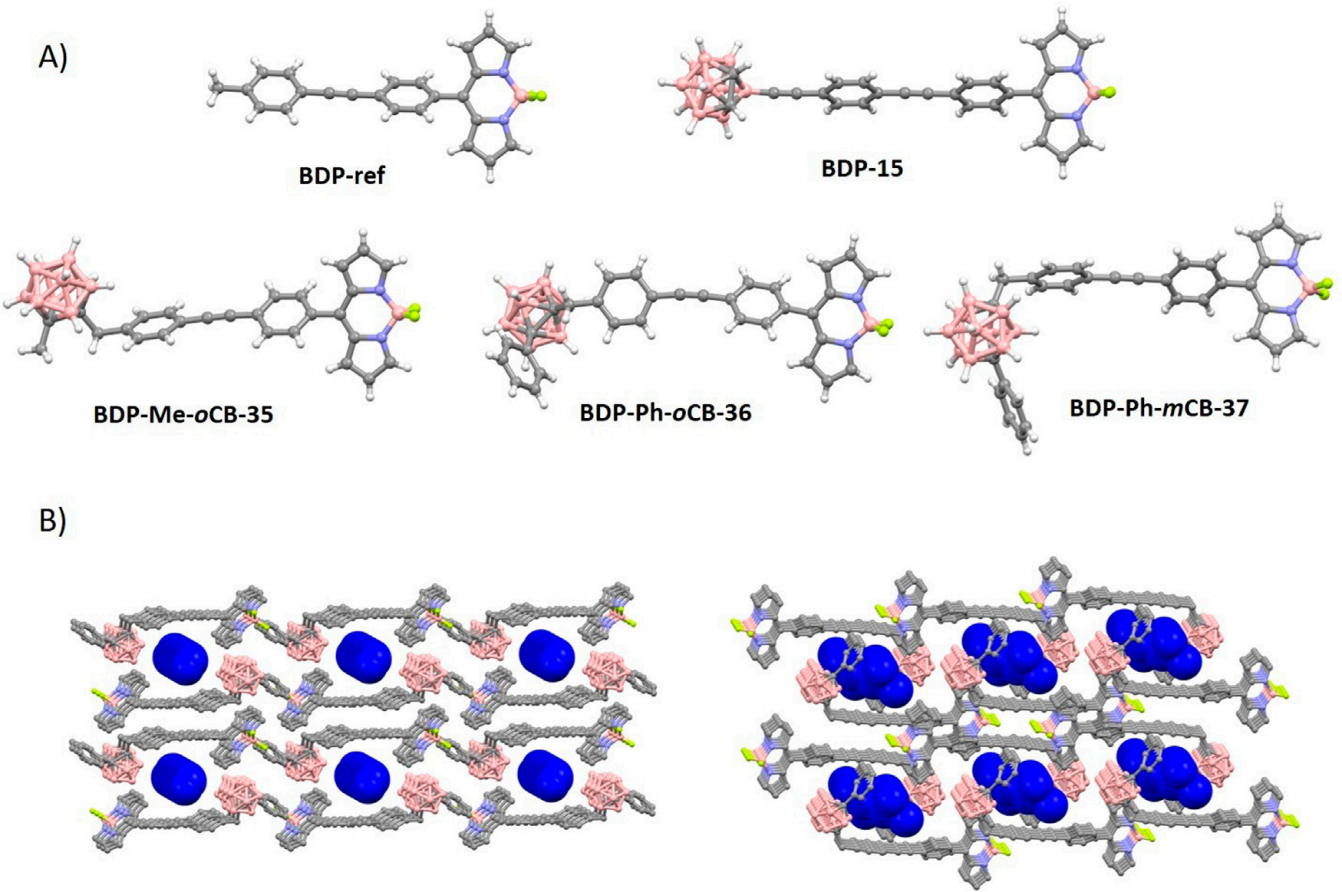
**(A)** Molecular structures for long carboranyl-aryl-*meso*-BODIPYs (Labra-Vázquez et al., 2020; Shlyakhtina et al., 2015). Color code: B, pink; C, grey; H, white; S, yellow; N, blue; and F, green. **(B)** Crystal packing views for **BDP-Ph-oCB-36** (left) and **BDP-Ph-mCB-37** (right) showing the disordered *n*-hexane in deep blue color. Other color codes: B, pink; C, grey; and F, green. H atoms are omitted for clarity.

The introduction of carboranyl moieties at the aryl-*meso* fragment does not appear to significantly affect the BODIPY structures (Figure 2A). The angles between the aryl-*meso* and BODIPY planes range from 50° to 89°, indicating limited rotation (Supplementary Table S10). As anticipated, a more perpendicular orientation is observed when the carborane moieties are bulkier and closer to the aryl-*meso* fragment. Generally, the quantum yields for this family of BODIPYs are relatively low, with only slight increases observed when carboranes are introduced (**BDP-ref** vs*.* **BDP-Me-*o*CB-35** and **BDP-Ph-*o*CB-36**).

The supramolecular structures of the BODIPY-carboranyl dyads **BDP-Ph-*o*CB-36** and **BDP-Ph-*m*CB-37** (Figure 2A) revealed intriguing arrangements within the crystal lattice (Labra-Vázquez et al., 2020). In the case of **BDP-Ph-*o*CB-36**, the solvent molecules (*n*-hexane) were confined within supramolecular pores formed by an antiparallel head-to-tail arrangement of pairs of fluorophores, extending along the crystallographic *a* axis (Figure 2B). This arrangement created distinct channels (5.2% of the unit cell) where the solvent molecules were encapsulated. On the other hand, for **BDP-Ph-*m*CB-37**, the *n*-hexane molecules were closely surrounded by the fluorophore molecules, indicating a more intimate interaction between the solvent and the fluorophores (Figure 2B). Unlike in the *ortho*-isomer, where the solvent molecules moved freely through supramolecular channels, in the *meta*-isomer, the solvent molecules were densely trapped within the crystal lattice. This difference in supramolecular organization suggests varying degrees of solvent-fluorophore interactions and highlights the influence of molecular structure on the overall packing arrangement within the crystals. Such compounds are, therefore, crystalline inclusion compounds. The awkward shape of the BODIPY-carboranyl dyads prevents efficient packing in the solid state, leading to the entrapment of molecular guests in their crystal lattice (Tsang et al., 2017).

### 3.4 BODIPY dyes functionalized with anionic *closo*-dodecaborate and metallacarboranes

**SCHEME 19:**
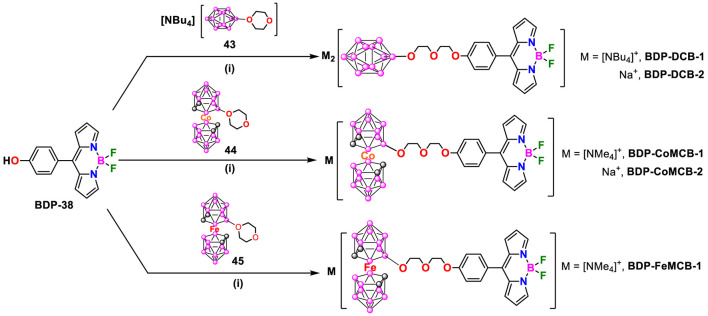
Synthesis of anionic boron clusters-based BODIPYs. (i) K_2_CO_3_, CH_3_CN.

R. Núñez and coworkers (Chaari et al., 2018a) reported a series of anionic boron cluster-BODIPY conjugated bearing *closo*-dodecaborate (**BDP-DCB-1**), cobaltabisdicarbollie (**BDP-CoMCB-1**) and ferrabisdicarbollide (**BDP-FeMCB-1**). These compounds exhibited excellent biological properties as fluorescent markers in cells and potential applications in BNCT (*vide infra*). They were synthesized by nucleophilic oxonium ring-opening reaction of [Bu_4_N][B_12_H_11_(C_4_H_8_O_2_)] (**43**), [3,3′-Co(8-C_4_H_8_O_2_-1,2-C_2_B_9_H_10_)(1′,2′-C_2_B_9_H_11_)] (**44**) and [3,3′-Fe (8-C_4_H_8_O_2_-1,2-C_2_B_9_H_10_)(1′,2′-C_2_B_9_H_11_)] (**45**) (Scheme 19). The first step was the deprotonation of the phenol group in **BDP-38** with K_2_CO_3_ in CH_3_CN followed by the nucleophilic attack to the oxonium ring. BODIPYs **BDP-DCB-1**, **BDP-CoMCB-1** and **BDP-FeMCB-1** were precipitated as [NBu_4_]^+^ or [NMe_4_]^+^salts in good yields, ranged from 68% to 88%. The corresponding sodium salts were obtained by cation exchange by using cation-exchange resin.

Absorption and emission properties were analyzed in THF solutions and for **BDP-DCB-2** also in water at room temperature. The UV-vis spectra for all of them showed a characteristic absorption maxima at around 497 nm (Supplementary Table S11), corresponding to the S0-S1 (π-π*) transitions in the BODIPY core. A shoulder at higher energy attributed to vibrational transitions was also present. The emission maxima were in the range from 504 to 520 nm due to the BODIPY with low QYs (3%–6%) and Stokes shift values from 7 to 23 nm. The link of these anionic boron clusters to the BODIPY did not significantly affect the absorption and emission properties of the BODIPY dye except for a slight decrease in the quantum efficiency.

### 3.5 Biological applications of boron cluster-BODIPY dyes

BNCT is a promise cancer therapy for the treatment of high-grade brain tumors, such as glioblastoma multiforme (GBM). For this purpose, one of the main challenges for the application of BNCT in brain tumors arises from the existence of the blood–brain barrier (BBB) that prevents most drugs from penetrating into the brain and from reaching the targeted tumor cells. Carborane-BODIPY derivatives were designed as potential boron delivery agents for BNCT. For this purpose, M.G. Vicente et al. (Gibbs et al., 2015) studied the BBB permeability of **BDP-Me-*o*CB-20** (Pe = 0.05 × 10^−6^ cm/s) and **BDP-Me-*o*CB-21** (Pe = 0.13 × 10^−6^ cm/s) in human brain endothelial cell line hCMEC/D3 to demonstrated that these BODIPYs were poorly internalized probably due to their high hydrophobicity (log P of 1.41 and 1.35, respectively).

The BODIPY derivatives **BDP-*o*CB-41** to **BDP-*o*CB-48** were also designed for potential use for BNCT. Examination of their BBB permeability in the human brain endothelial cell line hCMEC/D3 showed that they were effectively internalized. **BDP-*o*CB-41** showed higher BBB permeability (Pe = 39 × 10^−6^ cm/s) than Lucifer yellow (Pe = 25.23 × 10^−6^), probably as a result of its lower molecular weight and lower hydrophobic character (log *p* = 0.85) (Supplementary Table S12). These BODIPYs showed low dark toxicity and phototoxicity (IC50 > 80 μM) in human glioma T98G cells, with the exception of **BDP-*o*CB-45** (IC_50_ = 40 μM), probably as a result of its remarkably high uptake into cells. On the other hand, **BDP-*o*CB-42** showed the largest permeability (Pe = 164 × 10^−6^ cm/s) across the BBB model consisting of hCMEC/D3 cells. Results indicated that those BODIPYs with MW < 400 Da and log *p* < 1.7 are the most efficient at crossing the BBB model. The most hydrophobic compound **BDP-*o*CB-48** (log *p* = 2.70) was poorly soluble in aqueous solutions, showed very low uptake into T98G cells, as it was precipitated in buffer precluded determination of its BBB permeability. In general, BODIPYs tested showed higher BBB permeability compared with Lucifer yellow and lower dark cytoxicity, suggesting that they may be effective boron delivery agents for BNCT of brain tumors. Among this series, **BDP-*o*CB-42** and **BDP-*o*CB-43** showed the highest BBB permeability, while **BDP-*o*CB**-**45** and **BDP-*o*CB-46** were more accumulated within tumor cells; therefore, these are the most promising BNCT agents.

Compounds **BDP-*o*CB-53**, **BDP-*m*CB-54**, **BDP-*o*CB-55**, **BDP-*m*CB-26**, **BDP-monoCB-57** and **BDP-monoCB-58** exhibited single oxygen (^1^O_2_) production upon irradiation, being **BDP-*o*CB-53** the one with the highest ability to generate ^1^O_2_. Studies reported by A. Ol’shevskaya and coworkers indicated that non-methylated derivatives showed an increased non-radiative deactivation due to enhanced rotational freedom of *meso*-tetrafluorophenyl group. These compounds have lower fluorescence efficiency whereas the rate of intersystem crossing is increased. None of the compounds showed cytotoxic in HCT116 colon adenocarcinoma cells in the dark, whereas they potently induced lipid peroxidation and rapid (within minutes) cell death upon photoactivation. Compounds B**DP-*o*CB-53**, **BDP-monoCB-57** and **BDP-monoCB-58** formed stable complexes with Bovine Serum Albumin (BSA) as it has been demonstrated by the binding constants (Kb) that range from 1.1 to 3.5 × 10^−5^ M^−1^, property that can help the delivery of these dyes in the body. Compound **BDP-*o*CB-53** was localized in lysosomes and partially in mitochondria of HCT116 colon adenocarcinoma cells, whereas **BDP-monoCB-58** was visualized in cytoplasm, membrane and mitochondria, but not in lysosomes after incubation for 24 h. Data on lipid peroxidation in cells incubated with **BDP-*o*CB-53** or **BDP-monoCB-58** clearly demonstrated that the increased fluorescence of the oxidized form upon light excitation indicates massive lipid peroxidation that precedes the plasma membrane damage. The peroxidation of membrane lipids was higher for **BDP-monoCB-58** than for **BDP-*o*CB-53** and **BDP-monoCB-58** was a more potent inducer of necrosis than **BDP-oCB-53**. Carboranyl-BODIPY conjugates represent a new chemotype perspective for binary therapeutic modalities.

**FIGURE 3:**
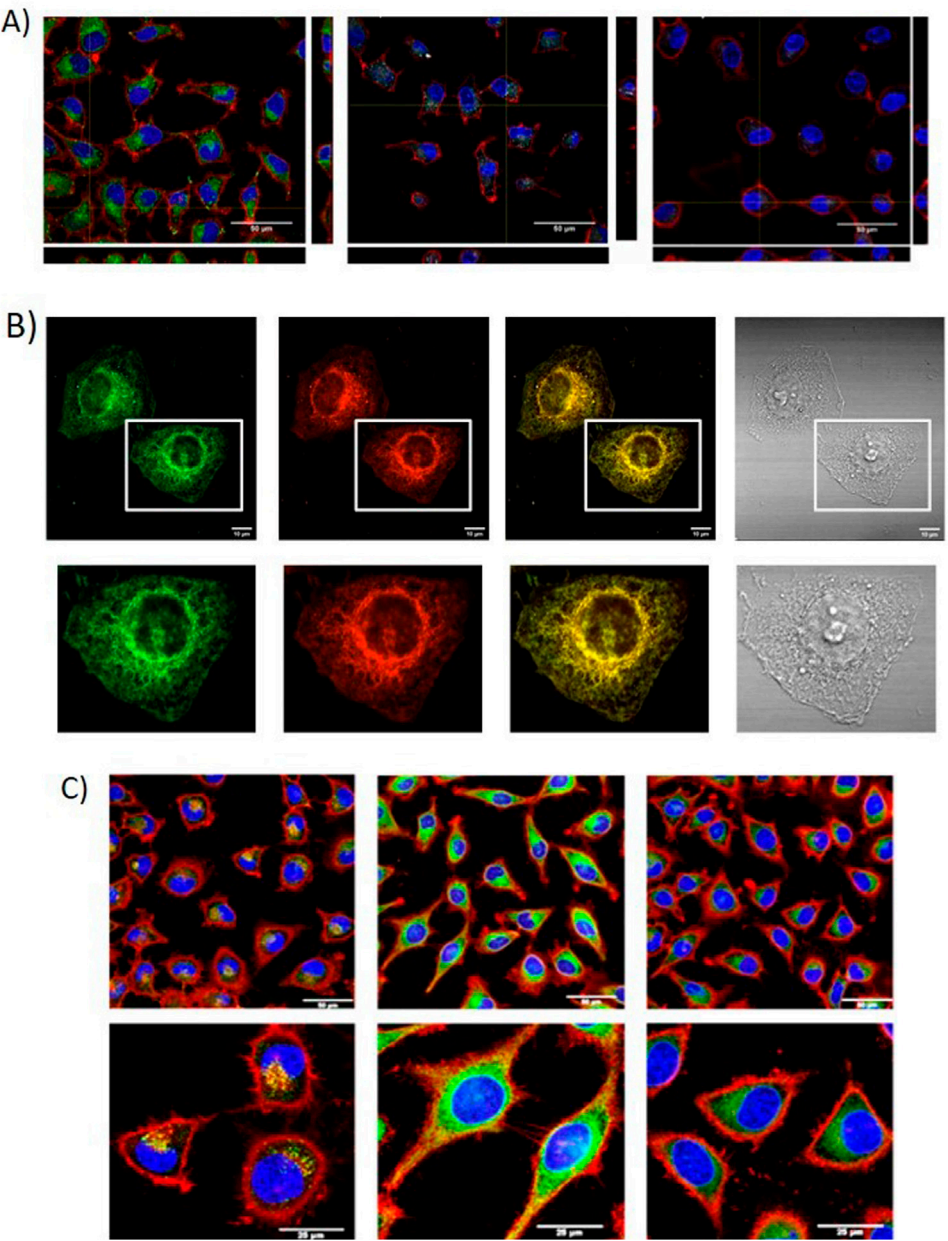
**(A)** Orthogonal projections of HeLa cells incubated with compounds **aza-BDP-Me-*o*CB-39** (grey), **aza-BDP-Ph-*o*CB-40** (grey) and **BDP-Me-*m*CB-24** (green) (scale bar: 50 µm). **(B)** Confocal images of 10 μM **BDP-*m*Ph-CB-37** in live HeLa cells after 30 min incubation. Panels from left to right show the confocal green and red channel, merged and bright channels, respectively (scale bars:13 μm. **(C)** Cellular uptake of **BDP-DCB-1**, **BDP-CoMCB-1** and **BDP-FeMCB-1** after 2 h of incubation by confocal microscopy (scale bar: 50 μm).

Careborane-BODIPYs **BDP-Me-*o*CB-23** to **BDP-Me-*m*CB-26** and carboranes-aza-BODIPY **aza-BDP-Me-mCB-38**, **aza-BDP-Me-*m*CB-39** and **aza-BDP-Ph-*m*CB-40** were studied as potential fluorescence probes for biological systems (Bellomo et al., 2018). All of them, except **aza-BDP-Me-*o*CB-38**, were detected by confocal microscopy indicating that they were internalized and accumulated in the cytoplasm (Figure 3A), suggesting that Me-*o*-carborane caused a negative effect on the permeability of the BODIPY. **BDP-Me-*o*CB-23** to **BDP-Me-*m*CB-26** were in the range of green fluorescence, **aza-BDP-Me-*m*CB-39** and **aza-BDP-Ph-*m*CB-40** were observed in the range of far-red (NIR region, which is outside the visible spectra of human eye), whereas compound **BDP-Me-*m*CB-26** showed a wide emission range, from red to far-red of the spectral region. Compounds **BDP-Me-*o*-CB-23**, **BDP-Ph-*m*CB-25** and **BDP-Me-*m*CB-26** showed exceptional fluorescence characteristics for cell imaging purposes. **BDP-Me-*o*CB-23**, **BDP-Ph-*m*CB-25** (and **BDP-Me-*o*CB-26** when detected in the visible red wavelength) would be the first choice when a simple or double labelling is necessary. In addition, compound **BDP-Me-*o*CB-26** with NIR emission, could be very useful to avoid overlapping emission, mainly when multiple fluorochromes are used in the same sample. Compounds **BDP-Me-*o*CB-35**, **BDP-Ph-*o*CB-36** and **BDP-Ph-*m*CB-37** showed different behavior in HeLa cells internalization and subcellular distribution that were related to their unlike static dipole moments and partition coefficients (Labra-Vázquez et al., 2020). The Ph-*m*-carborane derivative **BDP-Ph-*m*CB-37** showed the highest lipophilicity with a log *p* = 0.677 being the best internalized and localized in the plasma membrane (Figure 3B). This evidence provides a molecular design strategy for improving the prospective applications of BODIPY–carboranyl dyads as potential fluorescence imaging agents and boron carriers for BNCT.

Cellular uptake of BODIPYs **BDP-DCB-1**, **BODB-DCB-2**, **BDP-CoMCB-1**, **BDP-CoMCB’** and **BDP-FeMCB-1** in HeLa cells at different concentration confirmed the excellent internalization of them by cells, especially for those containing the cobaltabisdicarbollide, showing a notable cytoplasmic staining (Figure 3C). The cellular uptake was analyzed by flow cytometry and confocal microscopy, and both techniques indicated better internalization of the target boron cluster-BODIPY conjugates compared to the starting **BDP-38**. These results suggested that linking the boron cluster clearly produces a synergistic effect, aiding the BODIPY in crossing the cell membrane. Noticeably, these dyes, especially compounds **BDP-CoMCB-1** and **BDP-CoMCB-2**, could be considered excellent candidates as theranostic agents for fluorescence cell imaging (diagnosis) and boron delivery systems for BNCT.

## 4 Systems based on BODIPY-labeled *nido*-*o*-carboranes

In the last decade, using electricity as a redox agent has made it possible to conduct chemical reactions efficiently and environmentally friendly. Therefore, electrocatalysis has become a useful tool for the regioselective activation of CH. However, there are a few examples of B-H functionalization.

**SCHEME 20:**
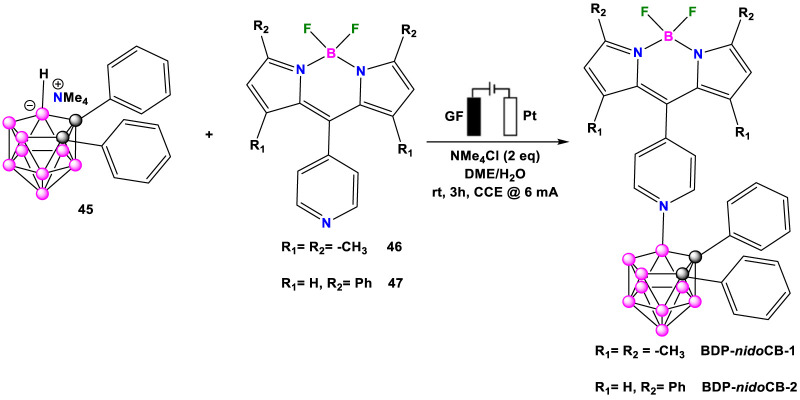
Synthesis of **BDP-*nido*CB-1** and **BDP-*nido*CB-2**.

In a groundbreaking development in 2021, Ackermann and his coworkers unveiled a novel strategy for the electrochemical regioselective cage B-H nitrogenation of *nido*-carborane **45** in a dehydrogenative manner, assembling a variety of N-heterocycles compounds, between them the BODIPY-labeled-pyridine **46** and **47** to obtain N-substituted *nido*-Carborane such as **BDP-*nido*CB-1** and **BDP-*nido*CB-2** (Scheme 20). (Yang et al., 2021) The electrochemical-catalyzed B-N reactions were carried out at room temperature in a simple undivided cell setup equipped with a GF (Graphite Felt) anode and a Pt-plate cathode. Two equivalents of NMe_4_Cl were used as an additive, and a mixture of 1,2-dimethoxyethane (DME)/H_2_O served as a solvent mixture.

The optical properties of **BDP-*nido*CB-1** and **BDP-*nido*CB-2** were studied by absorption and emission spectroscopy in five different solvents (Supplementary Table S13). Both compounds exhibited intense absorption. **BDP-*nido*CB-1** presented an absorption maxima band between 507–513 nm, while **BDP-*nido*CB-2** was between 570–582 nm. Both bands can be attributed to the S_1_←S_0_ (π-π*) transition of the BODIPY core. Additionally, both compounds displayed high Stokes shifts and molar extinction coefficients.

## 5 Summary and outlook

In conclusion, the integration of carborane structures into BODIPY dyes has led to significant advancements in the fields of photonics and materials science. The unique properties of carboranes, including their exceptional thermal stability, chemical robustness, and ability to enhance photophysical characteristics, have been effectively harnessed to create novel dye systems with superior performance. The studies presented herein highlight the efficient electronic energy transfer (EET) mechanisms facilitated by varying linker lengths, underscoring the delicate balance between structural design and functional efficiency. Notably, the findings indicate that while shorter linkers promote higher EET efficiencies, the introduction of longer spacers can lead to a decrease in energy transfer rates, thereby providing critical insights for future design strategies.

Looking ahead, the potential applications of carborane-based BODIPY dyes are vast and varied. Their enhanced photostability and tunable emission properties position them as promising candidates for use in advanced optoelectronic devices, including organic light-emitting diodes (OLEDs), solar cells, and sensors. Furthermore, the biocompatibility and selective targeting capabilities of these dyes incorporation carboranes open new avenues for their application in bioimaging and therapeutic modalities, particularly in the realm of cancer treatment through BNCT.

However, it is important to note that studies of the photophysical properties of carborane-BODIPY dyes in the solid state have been scarcely explored. This area presents a significant opportunity for future research, as understanding the behavior of these dyes in solid matrices could lead to the development of more efficient materials for various applications. Moreover, the ability of certain carborane-BODIPY systems to exhibit AIE could enhance their utility in responsive materials and solid-state luminescent devices, further broadening their application spectrum. Additionally, the potential of carborane-BODIPY dyes in antimicrobial and anticancer photodynamic therapy (PDT) remains largely untapped. Investigating their efficacy in these domains could yield valuable insights and innovative therapeutic strategies.

Future research endeavors should focus on the development of new synthetic methodologies to streamline the production of carborane-BODIPY derivatives, as well as the exploration of their mechanisms of action at the molecular level. Investigating the interactions between these dyes and biological systems will be crucial for optimizing their performance in medical applications. Additionally, the incorporation of other functional groups and materials could lead to the discovery of hybrid systems that leverage the strengths of both organic and inorganic components.

In summary, the ongoing exploration of carborane-based BODIPY dyes holds great promise for unlocking innovative applications across multiple fields. As research progresses, we anticipate that these compounds will not only enhance our understanding of photophysical processes but also contribute to the development of next-generation technologies that harness light in novel and impactful ways. The journey of carborane-BODIPY research is just beginning, and we are excited to witness the unfolding of its potential in the coming years.
