# Supplementary material for: Carborane-based BODIPY dyes: synthesis, structural analysis, photophysics and applications
Source: Front Chem. 2024 Nov 5;12:1485301. doi: 10.3389/fchem.2024.1485301 (PMC11574714; doi:10.3389/fchem.2024.1485301)
Supplement: Supplementary file 1 [file DataSheet1.pdf]

Carborane-Based BODIPY Dyes: Synthesis, Structural Analysis, Photophysics and Applications

Supporting Information

Javier Ordóñez-Hernández, José Giner Planas, Rosario Núñez*

Institut de Ciència de Materials de Barcelona (ICMAB-CSIC), Campus de la UAB, 08193, Bellaterra, Barcelona (Spain)

TABLE OF CONTENTS

Table S1. Photophysical properties of Ziessel´s D-A dyads…………………………...S2
Table S2. Photophysical properties and **BDP-18**, **BDP-19** and **BDP-*o*CB-12** and **BDP-*o*CB-13**…………………………………………………………………………….S3
Table S3. Optical properties of axle **BDP-23** and nanocars……………………………S3
Table S4. Photoluminesce (PL) behaviors of **BDP-*o*CB-17** and **BDP-*o*CB-18**……….S3
Table S5. Absorption and emission properties of carborane-BODIPY and aza-BODIPY dyes in THF at r.t……………………………………………………………………..S4
Table S6. Absorption and emission properties of BODIPYs at room temperature…….S5
Table S7. Photophysical properties of BODIPYs decorates with mercaptocarboranes in EtOH…………………………………………………………………………………S5
Table S8. Crystallographic parameters measured for *meso*-substituted carboranylthio BODIPY structures. See Figure 1a for the molecular structures and Chart 1 for nomenclature……………………………………………………………………….S6
Table S9. Crystallography parameters measured for *3/5*-substituted carboranylthio BODIPY structures. See Figure 1b for the molecular structures and Chart 1 for nomenclature…..S6
Table S10. Crystallography parameters measured for carboranyl-aryl BODIPY structures. See Figure 2 for the molecular structures and Chart 1 for nomenclature………………..S6
Table S11. Photophysical data for anionic boron clusters-BODIPY conjugates………..S7
Table S12. Permeability and Partition coefficient (Log P) of some compounds………..S8
Table S13. Spectroscopic data of **BDP-*nido*CB-1** and **BDP-*nido*CB 2**………………...S8
References…………………………………………………………………………….S9

Table S1. Photophysical properties of Ziessel´s D-A dyads. (Ziessel et al., 2010),(Hablot et al., 2012), (Harriman et al., 2013), (Hablot et al., 2013)

Compound	Donor	Acceptor	Linker	λ_{abs} (nm)	λ_{em} (nm)	QY	$k_{ET}(s^{-1})$
**BDP-*p*CB-1[a]**	BDP-1	BDP-2	*p*CB	694	728	0.30	$6.2x10^{8}$
**BDP-*p*-CB-1+H[+a]**	BDP-1	BDP-2	*p*CB	628	638	0.42	$1.7x10^{9}$
**BDP-*P*CB-2[b]**	DPP	SubPc	*p*CB	645	659	0.47	$1.7x10^{9}$
**BDP-pCB-3[b]**	DPP	BDP	*p*CB	645	659	0.47	$1.2x10^{10}$
**BDP-*P*CB-4[b]**	DPP	SubPc	$(p\text{CB})_2$	645	659	0.36	$3.2x10^{8}$
**BDP-*p*CB-5[c]**	DPP	BDP	*p*CB	672	687	N/R	$3.1x10^{9}$
**BDP-*o*CB-6[c]**	DPP	BDP	*o*CB	672	687	N/R	$1.9x10^{10}$
**BDP-*p*CB-2[c]**	DPP	BDP	*p*CB	645	662	0.10	$2.1x10^{9}$
**BDP-*p*CB-7[c]**	DPP	BDP	$(p\text{CB})_2$	645	662	0.44	$1.9x10^{8}$
**BDP-*p*CB-8[c]**	DPP	BDP	$(p\text{CB})_3$	645	662	0.73	$4.1x10^{7}$
**BDP-*p*CB-9[c]**	DPP	BDP	$(p\text{CB})_4$	645	662	0.81	$1.4x10^{7}$
**BDP-*p*CB-10[c]**	DPP	BDP	$(p\text{CB})_5$	645	662	0.84	$4.2x10^{6}$

[a]1,4-dioxane; [b]THF; [c]2-MTHF

Table S2. Photophysical properties and **BDP-18**, **BDP-19** and **BDP-*o*CB-12** and **BDP-*o*CB-13.**(Jin et al., 2015)

Compounds	λ_{abs} (nm)	ε_{max} ($M^{-1}cm^{-1}$)	λ_{em} (nm)[a]	τ_f (nm)[b]	QY[c]
**BDP-18**	526	158489	543	4.0	0.41
**BDP-19**	526	125893	543	3.8	0.45
**BDP-*o*CB-12**	531	100000	544	1.8	0.31
**BDP-*o*CB-13**	530	158489	544	1.4	0.23

[a]λ_{ex} = 500 nm, [b]λ_{ex} = 408 nm, [c]Reference: rhodamine B (QY= 0.49, 500 nm), λ_{ex} = 500 nm

Table S3. Optical properties of axle **BDP-23** and nanocars.(Godoy et al., 2010)

Compound	λ_{abs} (nm)	ε_{max} ($M^{-1}cm^{-1}$)	λ_{em} (nm)	QY[a]
**BDP-23**	552	116000	567	0.85
**BDP-*p*CB-14**	554	123000	571	0.69
**BDP-*p*CB-15**	552	181000	569	0.70
**BDP-*p*CB-16**	552	191000	567	0.79

[a]Determined in chloroform solution, ca $1x10^{-7}$ M. Using rhodamine 6G as reference, φ_F = 0.95 in EtOH, λ_{exc} 488 nm.

Table S4. Photoluminesce (PL) behaviors of **BDP-*o*CB-17** and **BDP-*o*CB-18**.(Tominaga et al., 2014)

Compound	In THF[a]		Aggregate[b]		Solid	
	λ_{em} (nm)	QY[c]	λ_{em} (nm)	QY[c]	λ_{PL} (nm)	QY[c]
**BDP-*o*CB-17**	512	0.33	533	0.02	563	<0.01
**BDP-*o*CB-18**	513	0.40	529	0.02	544	<0.01

[a]$1x10^{-5}$ M. [b]In THF/H_2O (v/v=1:99), $1x10^{-5}$ M. [c]Absolute PL quantum efficiency.

Table S5. Absorption and emission properties of carborane-BODIPY and aza-BODIPY dyes in THF at r.t.(Gibbs et al., 2015),(Xuan et al., 2016), (Bellomo et al., 2018), (Labra-Vázquez et al., 2020), (Bellomo et al., 2021).

**Compound**						
#	**CB Isomer**	**λ_{abs} (nm)**	**λ_{em} (nm)**	**Log ε ($M^{-1}cm^{-1}$)**	**QY**	**Stokes shifts (nm)**
**BDP-Me-*o*CB-20[a]**	*oCB*	534	561	4.38	0.99	27
**BDP-Me-*o*CB-21[a]**	*oCB*	501	508	4.81	0.99	7
**BDP-Me-*o*CB-22[a]**	*oCB*	502	517	4.50	0.034	15
**BDP-Me-*o*CB-23[b]**	*oCB*	501	512	4.89	0.404	11
**BDP-Me-*m*CB-24[b]**	*mCB*	501	511	4.91	0.403	10
**BDP-Ph-*m*CB-25[b]**	*mCB*	501	511	4.92	0.435	10
**BDP-Me-*m*CB-26[b]**	*mCB*	618	641	4.82	0.49	23
**BDP-Me-*m*CB-27[c]**	*mCB*	584	640	4.75	0.14	56
**BDP-Ph-*m*CB-28[c]**	*mCB*	578	643	4.54	0.11	65
**BDP-Ph-*m*CB-29[c]**	*mCB*	580	640	4.46	0.14	60
**BDP-Me-*m*CB-Ph-*o*CB-32[c]**	*mCB, oCB*	578	643	4.69	0.12	65
**BDP-Me-*m*CB-Ph-mCB-33[c]**	*mCB, mCB-*	582	641	4.76	0.12	59
**BDP-Ph-*m*CB-34[c]**	*mCB*	641	651	4.95	0.36	10
**BDP-Me-*o*CB-35[d]**	*oCB*	501	520	5.25	0.011	19
**BDP-Ph-*o*CB-36[d]**	*oCB*	501	520	4.84	0.012	19
**BDP-Ph-*m*CB-37[d]**	*mCB*	501	521	5.21	0.006	20
**aza-BDP-Me-*o*CB-38[b]**	*oCB*	665	705	4.86	0.015	40
**aza-BDP-Me-*m*CB-39[b]**	*mCB*	665	706	4.91	0.014	41
**aza-BDP-Ph-*o*CB-40[b]**	*oCB*	665	707	4.91	0.014	42

[a]Measured in DMSO; [b]Rhodamine 6G in ethanol (QY = 0.95) (Fischer and Georges, 1996) was used as standard; [c]Rhodamine 101 in methanol (QY = 1) as standard (Brouwer, 2011); [d]Rhodamine B as standard in ethanol (QY = 0.5). (Nostrand et al., 1980)

Table S6. Absorption and emission properties of BODIPYs at room temperature. (Wang et al., 2014),(Gibbs et al., 2015), (Zhao et al., 2015), (Xuan et al., 2016)

Compound						
#	CB Isomer	λ_{abs} (nm)	λ_{em} (nm)	QY[a]	Log ε ($M^{-1}cm^{-1}$)	Stokes Shifts (nm^{-1})
**BDP-*o*CB-41**[b]	*oCB*	538	n/a	0.01	4.37	
**BDP-*o*CB-42**	*oCB*	544	556	0.52	4.42	12
**BDP-*o*CB-43**	*oCB*	537	553	0.29	4.54	16
**BDP-*o*CB-44**	*oCB*	554	576	0.065	4.45	22
**BDP-*o*CB-45**	*oCB*	577	608	0.060	4.29	31
**BDP-*o*CB-46**	*oCB*	582	609	0.090	4.16	27
**BDP-*p*CB-47**[b]	*pCB*	531	545	0.03	4.68	14
**BDP-*o*CB-48**	*oCB*	521	540	0.58	4.18	19

[a]Rhodamine 6G in ethanol (0.95) was used as standard for all compounds except for **BDP-*o*CB-42** and **BDP-*o*CB-43**, for which violet perchlorate in methanol (0.54) as the standard was used. [b]Measured in DMSO

Table S7. Photophysical properties of BODIPYs decorates with mercaptocarboranes in EtOH. (Zaitsev et al., 2022a), (Zaitsev et al., 2022b), (Zaitsev et al., 2023).

Compound						
#	CB Isomer	λ_{abs} (nm)	λ_{em} (nm)	QY	ε ($M^{-1}cm^{-1}$)	Stokes Shifts (nm)
**BDP-*o*CB-53**	*oCB*	515	538	0.63	34500	23
**BDP-*o*CB-54**	*oCB*	514	530	1	44380	16
**BDP-*m*CB-55**	*mCB*	513	538	0.62	41060	25
**BDP-*m*CB-56**	*mCB*	513	528	0.97	68310	15
**BDP-monoCB-57**	*monoCB-*	514	538	0.66	26850	24
**BDP-monoCB-58**	*monoCB*	513	538	1	44410	25
**BDP-*o*CB-59**	*oCB*	593	612	1	75500	19
**BDP-*m*CB-60**	*mCB*	596	615	1	77300	19
**BDP-monoCB-61**	*monoCB*	607	625	0.89	82000	18
**BDP-*o*CB-62**	*oCB*	593	615	1	80700	22
**BDP-*m*CB-63**	*mCB*	595	618	0.97	85600	23
**BDP-monoCB-64**	*monoCB*	607	632	0.91	83900	25

Table S8. Crystallographic parameters measured for *meso*-substituted carboranylthio BODIPY structures. See Figure 1a for the molecular structures and Chart 1 for nomenclature.

BODIPY	Torsion Angle (º) C=C–S–Cc	QY (solvent)
**BDP-*o*CB-41**	88.7	0.065 (DMSO)
**BDP-*o*CB-43**	93.9	0.29 (CH_2Cl_2)
**BDP-*o*CB-45**	86.2	0.060 (CH_2Cl_2)
**BDP-*p*CB-47**	99.6	0.03 (DMSO)

Table S9. Crystallography parameters measured for *3/5*-substituted carboranylthio BODIPY structures. See Figure 1b for the molecular structures and Chart 1 for nomenclature.

BODIPY	Pyrrole to CB Torsion Angles (º)	QY (solvent)
**BDP-*o*CB-48**	C(6)–C(5)–S–Cc: -86.4	0.58 (CH_2Cl_2)
**BDP-*m*CB-60**	C(2)–C(3)–S–B: 10.0<br>C(6)–C(5)–S–B: -9.0	1.0 (acetone)
**BDP-*m*CB-63**	C(2)–C(3)–S–B: 15.96<br>C(6)–C(5)–S–B: -9.0	1.0 (acetone)
**BDP-*o*CB-NUXKID***[*]	C(2)–C(3)–C–S: 84.7<br>C(6)–C(5)–S–Cc: -90.3	-

[*] Ning Zhao, M. Graca H. Vicente, F. R. Froncsek, CSC Communication (Private Communication), 2020, CCDC 2017688.

Table S10. Crystallography parameters measured for carboranyl-aryl BODIPY structures. See Figure 2 for the molecular structures and Chart 1 for nomenclature.

BODIPY	Meso-aryl group to BODIPY plane Angles (º)	QY (solvent)
**BDP-ref**	56.06	0.007 (EtOH)
**BDP-Me-*o*CB-35**	52.64	0.011 (EtOH)
**BDP-Ph-*o*CB-36**	50.26	0.012 (EtOH)
**BDP-Ph-*m*CB-37**	58.07	0.006 (EtOH)
**BDP-15**	59.13-60.7	-
**BDP-*m*CB-60**	63.51-66.37	1.0 (acetone)
**BDP-*m*CB-63**	65.39	1.0 (acetone)

Table S11. Photophysical data for anionic boron clusters-BODIPY conjugates.(Chaari et al., 2018)

Compound	λ_{abs} (nm)	$\varepsilon/10^5$ (M^{-1} cm^{-1})	λ_{em}(nm)	QY[a]	Stokes shift (nm)
**BDP-DCB-1**	496	0.536	517	0.06	21
**BDP-DCB-2**	497	0.495	516	0.06	19
**BDP-DCB-2**[b]	494	0.350	513	0.03	19
**BDP-CoMCB-1**	497	0.582	520	0.04	23
**BDP-CoMCB-2**	497	0.433	519	0.04	22
**BDP-FeMCB-1**	497	0.560	504	0.03	7

[a]Reference compound Rhodamine 6G (EtOH, QY = 0.94); [b]in water

Table S12. Permeability and Partition coefficient (Log P) of some compounds.(Wang et al., 2014), (Gibbs et al., 2015), (Zhao et al., 2015), (Xuan et al., 2016)

**Compound**	**Pe 10^{-6} (cm/s)**	**Log P**
**BDP-*o*CB-41**	38.75	0.85
**BDP-*o*CB-42**	164	1.50
**BDP-*o*CB-43**	60	1.69
**BDP-*o*CB-44**	44	1.63
**BDP-*o*CB-45**	26	1.95
**BDP-*o*CB-46**	54	1.93
**BDP-*p*CB-47**	0.38	0.89
**BDP-*o*CB-48**	-	2.70

Table 13. Spectroscopic data of **BDP-*nido*CB-1** and **BDP-*nido*CB 2**

Compound	Solvent	$^{Max}\lambda_{abs}$ [nm]	$^{Max}\lambda_{em}$ [nm]	Stokes shift [cm^{-1}]	ε_{max} [$M^{-1}cm^{-1}$]
**BDP-*nido*CB-1**	DCM	512	569	1956	68812
	$CHCl_3$	513	567	1856	69790
	Acetone	507	559	1834	73746
	DMF	509	561	1821	69557
	THF	509	562	1852	72549
**BDP-*nido*CB-2**	DCM	577	634	1558	63459
	$CHCl_3$	582	640	1557	60882
	Acetone	570	623	1492	65681
	DMF	574	602	810	58557
	THF	575	630	1518	67718

References

Bellomo, C., Chaari, M., Cabrera-González, J., Blangetti, M., Lombardi, C., Deagostino, A., et al. (2018). Carborane-BODIPY Dyads: New Photoluminescent Materials through an Efficient Heck Coupling. *Chem. - A Eur. J.* 24, 15622–15630. doi: 10.1002/chem.201802901

Bellomo, C., Zanetti, D., Cardano, F., Sinha, S., Chaari, M., Fin, A., et al. (2021). Red light-emitting Carborane-BODIPY dyes: Synthesis and properties of visible-light tuned fluorophores with enhanced boron content. *Dye. Pigment.* 194. doi: 10.1016/j.dyepig.2021.109644

Brouwer, A. M. (2011). Standards for photoluminescence quantum yield measurements in solution (IUPAC technical report). *Pure Appl. Chem.* 83, 2213–2228. doi: 10.1351/PAC-REP-10-09-31

Chaari, M., Gaztelumendi, N., Cabrera-González, J., Peixoto-Moledo, P., Viñas, C., Xochitiotzi-Flores, E., et al. (2018). Fluorescent BODIPY-Anionic Boron Cluster Conjugates as Potential Agents for Cell Tracking. *Bioconjug. Chem.* 29, 1763–1773. doi: 10.1021/acs.bioconjchem.8b00204

Fischer, M., and Georges, J. (1996). Fluorescence quantum yield of rhodamine 6G in ethanol as a function of concentration using thermal lens spectrometry. *Chem. Phys. Lett.* 260, 115–118. doi: 10.1016/0009-2614(96)00838-X

Gibbs, J. H., Wang, H., Bhupathiraju, N. V. S. D. K., Fronczek, F. R., Smith, K. M., and Vicente, M. G. H. (2015). Synthesis and properties of a series of carboranyl-BODIPYs. *J. Organomet. Chem.* 798, 209–213. doi: 10.1016/j.jorganchem.2015.05.009

Godoy, J., Vives, G., and Tour, J. M. (2010). Synthesis of highly fluorescent BODIPY-based nanocars. *Org. Lett.* 12, 1464–1467. doi: 10.1021/ol100108r

Hablot, D., Sutter, A., Retailleau, P., and Ziessel, R. (2012). Unsymmetrical p-carborane backbone as a linker for donor-acceptor dyads. *Chem. - A Eur. J.* 18, 1890–1895. doi: 10.1002/chem.201103307

Hablot, D., Ziessel, R., Alamiry, M. A. H., Bahraidah, E., and Harriman, A. (2013). Nanomechanical properties of molecular-scale bridges as visualised by intramolecular electronic energy transfer. *Chem. Sci.* 4, 444–453. doi: 10.1039/c2sc21505e

Harriman, A., Alamiry, M. A. H., Hagon, J. P., Hablot, D., and Ziessel, R. (2013). Through-space electronic energy transfer across proximal molecular dyads. *Angew. Chemie - Int. Ed.* 52, 6611–6615. doi: 10.1002/anie.201302081

Jin, G. F., Cho, Y. J., Wee, K. R., Hong, S. A., Suh, I. H., Son, H. J., et al. (2015). BODIPY functionalized o-carborane dyads for low-energy photosensitization. *Dalt. Trans.* 44, 2780–2787. doi: 10.1039/c4dt03123g

Labra-Vázquez, P., Flores-Cruz, R., Galindo-Hernández, A., Cabrera-González, J., Guzmán-Cedillo, C., Jiménez-Sánchez, A., et al. (2020). Tuning the Cell Uptake and Subcellular Distribution in BODIPY–Carboranyl Dyads: An Experimental and Theoretical Study. *Chem. - A Eur. J.* 26, 16530–16540. doi: 10.1002/chem.202002600

Nostrand, V., Hopflnger, A., Verlag, S., Rubber, C., and Correo, C. De (1980). But This. 1871–1872.

Tominaga, M., Naito, H., Morisaki, Y., and Chujo, Y. (2014). Control of the Emission Behaviors of Trifunctional o-Carborane Dyes. *Asian J. Org. Chem.* 3, 624–631. doi: 10.1002/ajoc.201300280

Wang, H., Vicente, M. G. H., Fronczek, F. R., and Smith, K. M. (2014). Synthesis and transformations of 5-chloro-2,2′-dipyrrins and their boron complexes, 8-chloro-BODIPYs. *Chem. - A Eur. J.* 20, 5064–5074. doi: 10.1002/chem.201304310

Xuan, S., Zhao, N., Zhou, Z., Fronczek, F. R., and Vicente, M. G. H. (2016). Synthesis and in Vitro Studies of a Series of Carborane-Containing Boron Dipyrromethenes (BODIPYs). *J. Med. Chem.* 59, 2109–2117. doi: 10.1021/acs.jmedchem.5b01783

Zaitsev, A. V., Kiselev, S. S., Smol’yakov, A. F., Fedorov, Y. V., Kononova, E. G., Borisov, Y. A., et al. (2023). BODIPY derivatives modified with carborane clusters: synthesis, characterization and DFT studies. *Org. Biomol. Chem.* 21, 4084–4094. doi: 10.1039/D3OB00255A

Zaitsev, A. V., Kononova, E. G., Markova, A. A., Shibaeva, A. V., Kostyukov, A. A., Egorov, A. E., et al. (2022a). A straightforward approach to carborane-substituted BODIPY derivatives via nucleophilic aromatic substitution: Synthesis and photodynamic properties. *Dye. Pigment.* 207, 110711. doi: 10.1016/j.dyepig.2022.110711

Zaitsev, A. V., Morozyuk, A. S., Kononova, E. G., Smol’yakov, A. F., and

Ol’shevskaya, V. A. (2022b). Maleimide-functionalized BODIPY dyes: Synthesis, structural determination and conjugation with carborane thiols. *J. Porphyr. Phthalocyanines* 26, 741–747. doi: 10.1142/S1088424622500596

Zhao, N., Vicente, M. G. H., Fronczek, F. R., and Smith, K. M. (2015). Synthesis of 3,8-Dichloro-6-ethyl-1,2,5,7-tetramethyl-BODIPY from an Asymmetric Dipyrroketone and Reactivity Studies at the 3,5,8-Positions. *Chem. - A Eur. J.* 21, 6181–6192. doi: 10.1002/chem.201406550

Ziessel, R., Ulrich, G., Olivier, J. H., Bura, T., and Sutter, A. (2010). Carborane-Bodipy scaffolds for through space energy transfer. *Chem. Commun.* 46, 7978–7980. doi: 10.1039/c0cc02656e
